# Supplementary material for: Synthesis of Novel 1-Oxo-2,3,4-trisubstituted Tetrahydroisoquinoline Derivatives, Bearing Other Heterocyclic Moieties and Comparative Preliminary Study of Anti-Coronavirus Activity of Selected Compounds
Source: Molecules. 2023 Feb 3;28(3):1495. doi: 10.3390/molecules28031495 (PMC9921785; doi:10.3390/molecules28031495)
Supplement: Supplementary file 1 [file molecules-28-01495-s001.zip › molecules-2134984-supplementary.pdf]

Supplementary materials for

Synthesis of Novel 1-Oxo-2,3,4-trisubstituted Tetrahydroisoquinoline Derivatives, Bearing Other Heterocyclic Moieties and Comparative Preliminary Study of Anti-coronavirus Activity of Selected Compounds

Meglena I. Kandinska[1], Nikola T. Burdzhiev[1,*], Diana V. Cheshmedzhieva[1], Sonia V. Ilieva[1], Peter P. Grozdanov[2], Neli Vilhelmova-Ilieva[2], Nadya Nikolova[2], Vesela V. Lozanova[3] and Ivanka Nikolova[2,*]

Table of Contents

Spectra of *Rel*-(3R,4R)-3-(1*H*-indol-3-yl)-2-(2-methoxyethyl)-1-oxo-1,2,3,4-tetrahydroisoquinoline-4-carboxylic acid (**3**) 2

Spectra of *Rel*-(3R,4R)-3-(1*H*-indol-3-yl)-*N*-isopropyl-*N*-(isopropylcarbamoyl)-2-(2-methoxyethyl)-1-oxo-1,2,3,4-tetrahydroisoquinoline-4-carboxamide (**4a**) 5

Spectra of *Rel*-(3R,4R)-*N*-isopropyl-*N*-(isopropylcarbamoyl)-2-(2-methoxyethyl)-1-oxo-3-(1-(piperidin-1-ylmethyl)-1*H*-indol-3-yl)-1,2,3,4-tetrahydroisoquinoline-4-carboxamide (**4b**) 10

Spectra of *Rel*-(3R,4R)-3-(1*H*-indol-3-yl)-2-(2-methoxyethyl)-4-(morpholine-4-carbonyl)-3,4-dihydroisoquinolin-1(2*H*)-one (**4c**) 15

Spectra of *Rel*-(3R,4R)-4-(1*H*-imidazole-1-carbonyl)-3-(1*H*-indol-3-yl)-2-(2-methoxyethyl)-3,4-dihydroisoquinolin-1(2*H*)-one (**4d**) 18

Spectra of *Rel*-(3R,4R)-3-(1*H*-indol-3-yl)-2-(2-methoxyethyl)-4-(4-methylpiperazine-1-carbonyl)-3,4-dihydroisoquinolin-1(2*H*)-one (**4e**) 20

Spectra of *Rel*-(3*R*,4*R*)- and *rel*-(3*S*,4*R*)-2-Hexyl-1-oxo-3-(pyridin-2-yl)-1,2,3,4-tetrahydroisoquinoline-4-carboxylic acids (*trans*-**6** and *cis*-**6**) 23

Spectra of *Rel*-(3*R*,4*R*)-2-Hexyl-4-(hydroxymethyl)-3-(pyridin-2-yl)-3,4-dihydroisoquinolin-1(2*H*)-one (*trans*-**8**) 24

Spectra of *Rel*-2-(((3*S*,4*R*)-2-hexyl-1-oxo-3-(pyridin-2-yl)-1,2,3,4-tetrahydroisoquinolin-4-yl)methyl)isoindoline-1,3-dione (*trans*-**9**) 26

Spectra of *Rel*-(3*R*,4*S*)-4-(Aminomethyl)-2-hexyl-3-(pyridin-2-yl)-3,4-dihydroisoquinolin-1(2*H*)-one (*trans*-**10**) 28

Spectra of (*S*)-*N*-((3*R*,4*S*)-2-hexyl-1-oxo-3-(pyridin-2-yl)-1,2,3,4-tetrahydroisoquinolin-4-yl)methyl)-3-phenyl-2-(2,2,2-trifluoroacetamido)propanamide and (*S*)-*N*-((3*S*,4*R*)-2-hexyl-1-oxo-3-(pyridin-2-yl)-1,2,3,4-tetrahydroisoquinolin-4-yl)methyl)-3-phenyl-2-(2,2,2-trifluoroacetamido)propanamide (*trans*-**11a**+*trans*-**11b**) 29

Spectra of *Tert*-Butyl (*S*)-2-(((3*R*,4*S*)-2-hexyl-1-oxo-3-(pyridin-2-yl)-1,2,3,4-tetrahydroisoquinolin-4-yl)methyl)carbamoyl)pyrrolidine-1-carboxylate and *tert*-butyl (*S*)-2-(((3*S*,4*R*)-2-hexyl-1-oxo-3-(pyridin-

2-yl)-1,2,3,4-tetrahydroisoquinolin-4-yl)methyl)carbamoyl)pyrrolidine-1-carboxylate (*trans*-**12a**+trans-**12b**) 31

(*S*)-*N*-(((3*R*,4*S*)-2-hexyl-1-oxo-3-(pyridin-2-yl)-1,2,3,4-tetrahydroisoquinolin-4-yl)methyl)-4-(methylthio)-2-(2,2,2-trifluoroacetamido)butanamide and (*S*)-*N*-(((3*S*,4*R*)-2-hexyl-1-oxo-3-(pyridin-2-yl)-1,2,3,4-tetrahydroisoquinolin-4-yl)methyl)-4-(methylthio)-2-(2,2,2-trifluoro-acetamido)butanamide (*trans*-**13a**+*trans*-**13b**) 33

Optimized geometry at PCM/M06-2X/6-31+G(d,p) level of theory (solvent dichloroethane) of trans-6a' 34

Spectra of *Rel*-(3R,4R)-3-(1*H*-indol-3-yl)-2-(2-methoxyethyl)-1-oxo-1,2,3,4-tetrahydroisoquinoline-4-carboxylic acid (**3**)

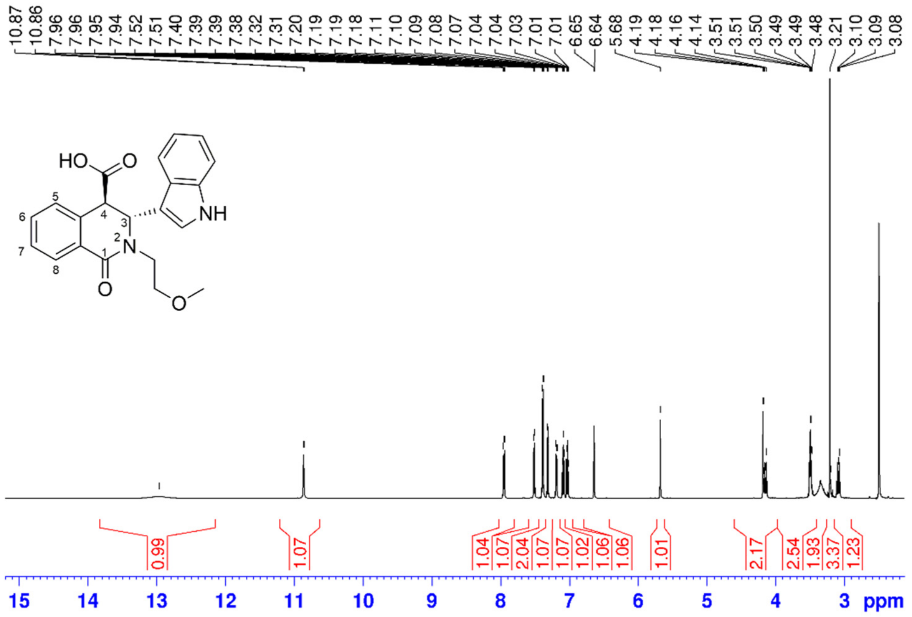

Figure S1. ^{1}H NMR (DMSO-d_6) of acid *trans*-**3** at 298 K.

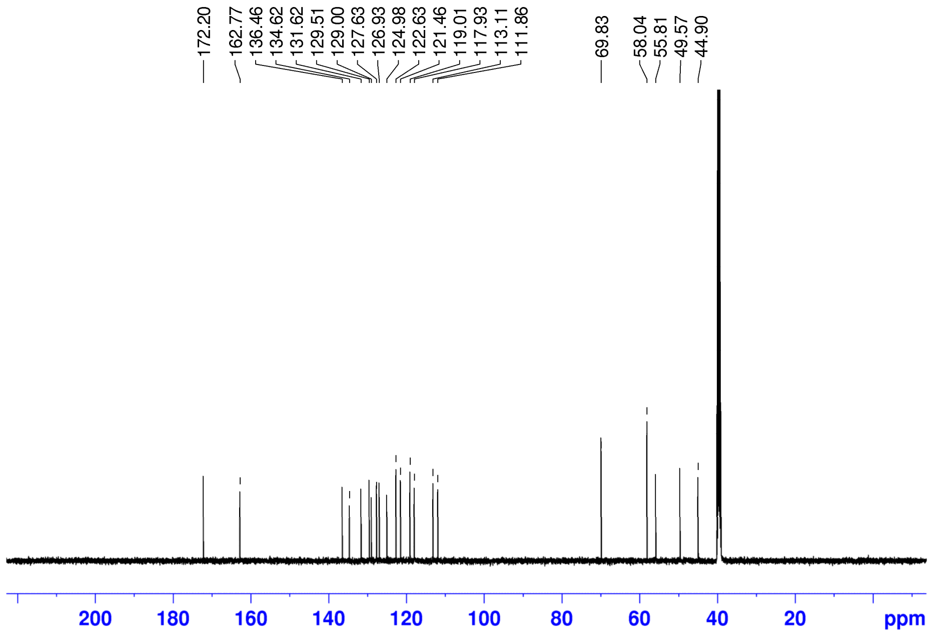

Figure S2. ^{13}C NMR (DMSO-d_6) of acid *trans*-**3** at 298 K.

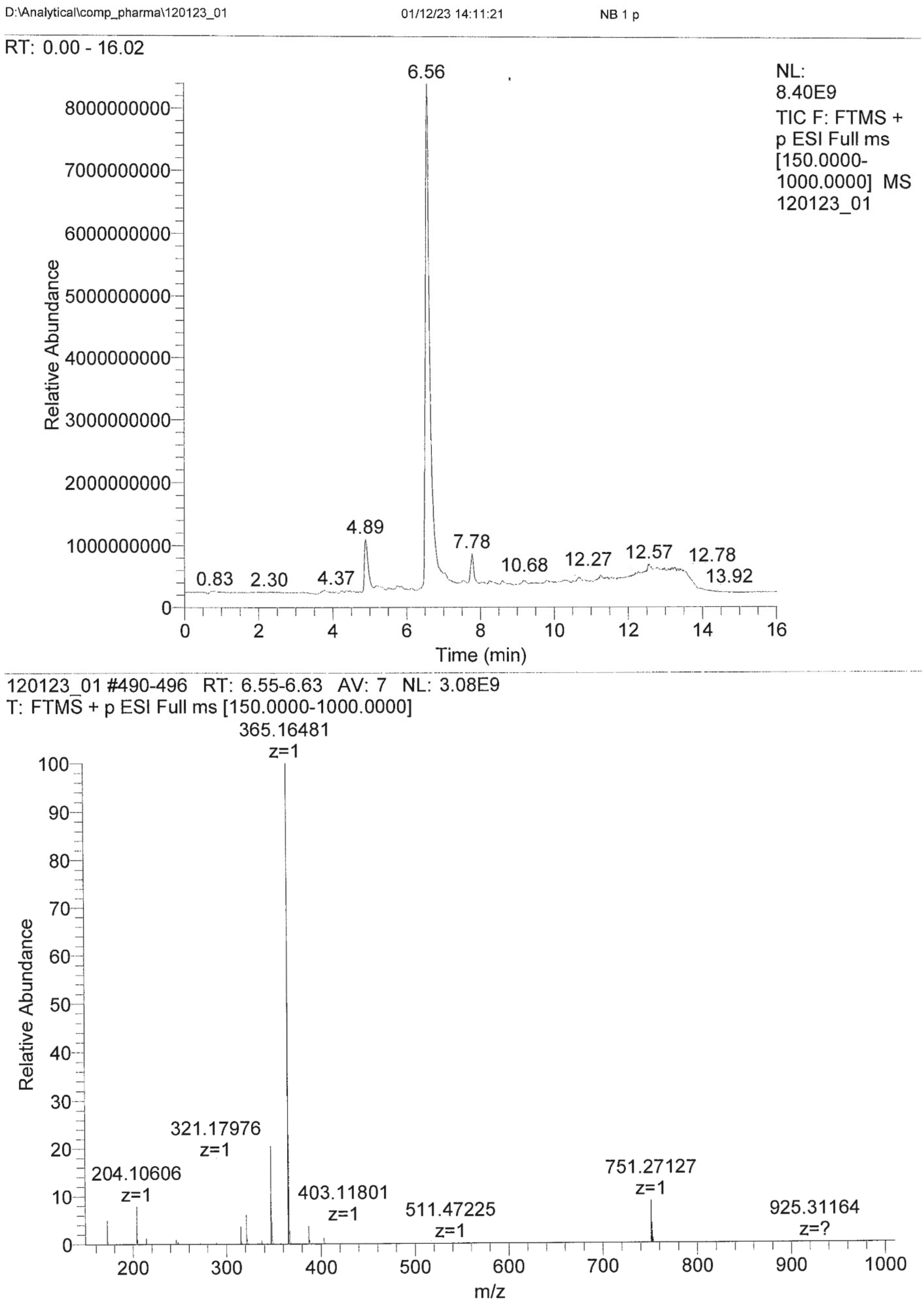

Figure S3. HRMS spectrum of acid *trans*-**3**.

Spectra of *Rel*-(3R,4R)-3-(1*H*-indol-3-yl)-*N*-isopropyl-*N*-(isopropylcarbamoyl)-2-(2-methoxyethyl)-1-oxo-1,2,3,4-tetrahydroisoquinoline-4-carboxamide (**4a**)

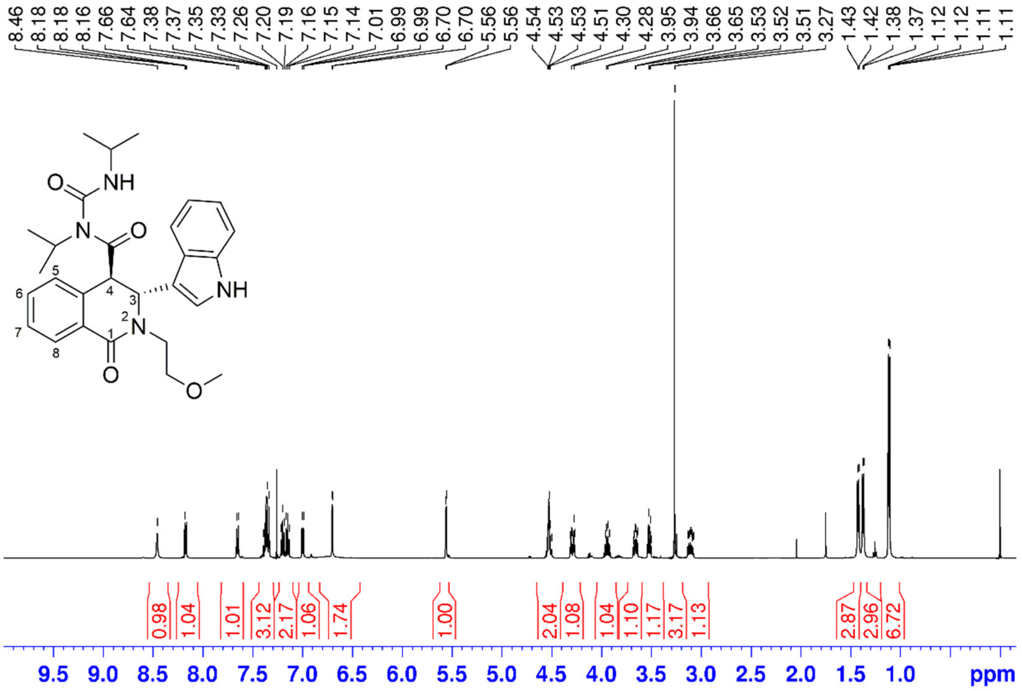

Figure S4. ^{1}H NMR ($CDCl_3$) of **4a** at 298 K.

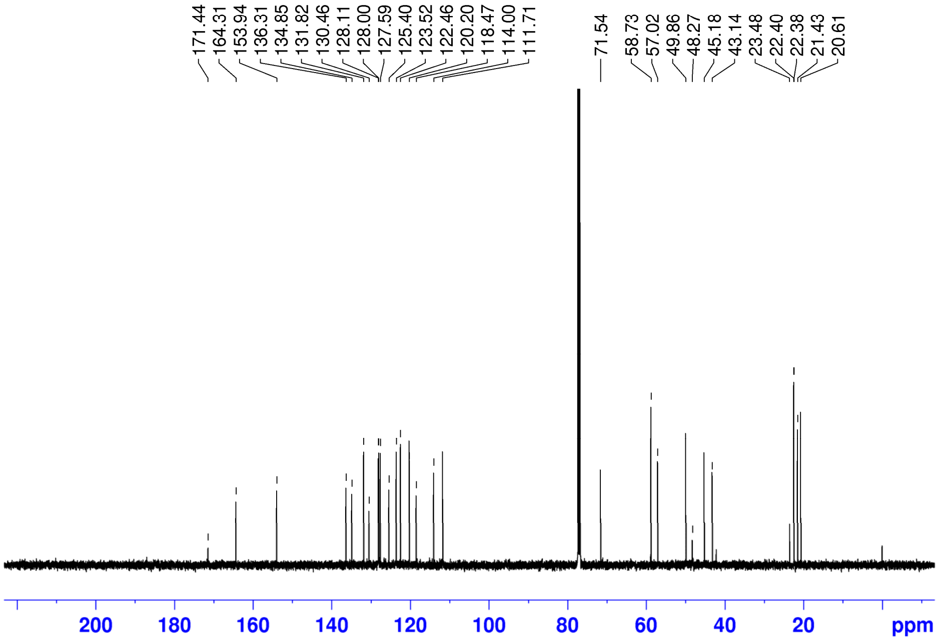

Figure S5. ^{13}C NMR ($CDCl_3$) of **4a** at 298 K.

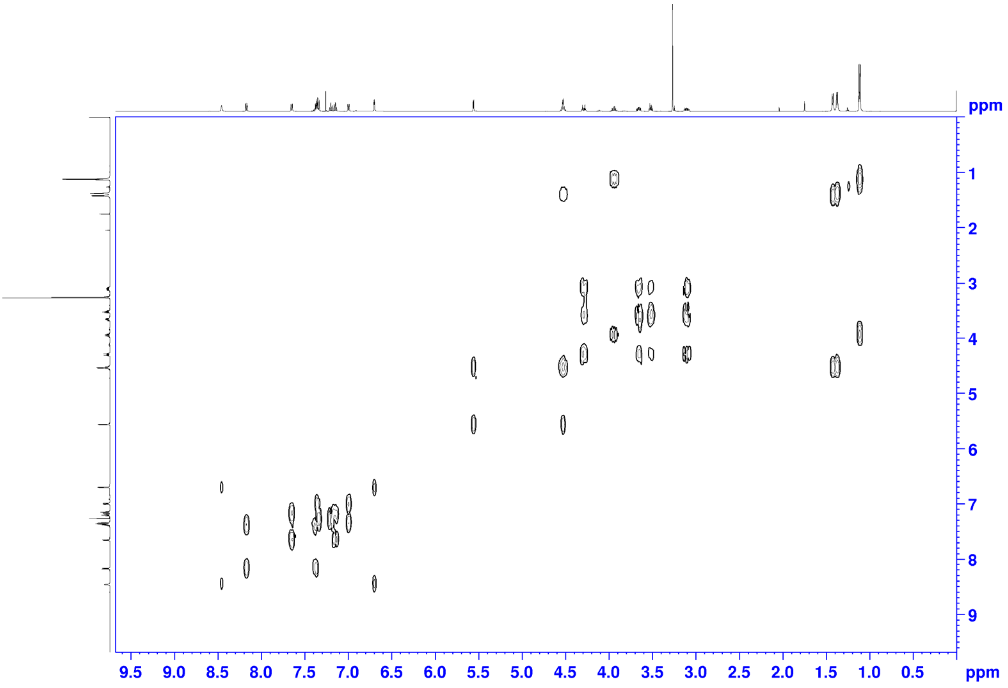

Figure S6. COSY NMR ($CDCl_3$) of **4a** at 298 K.

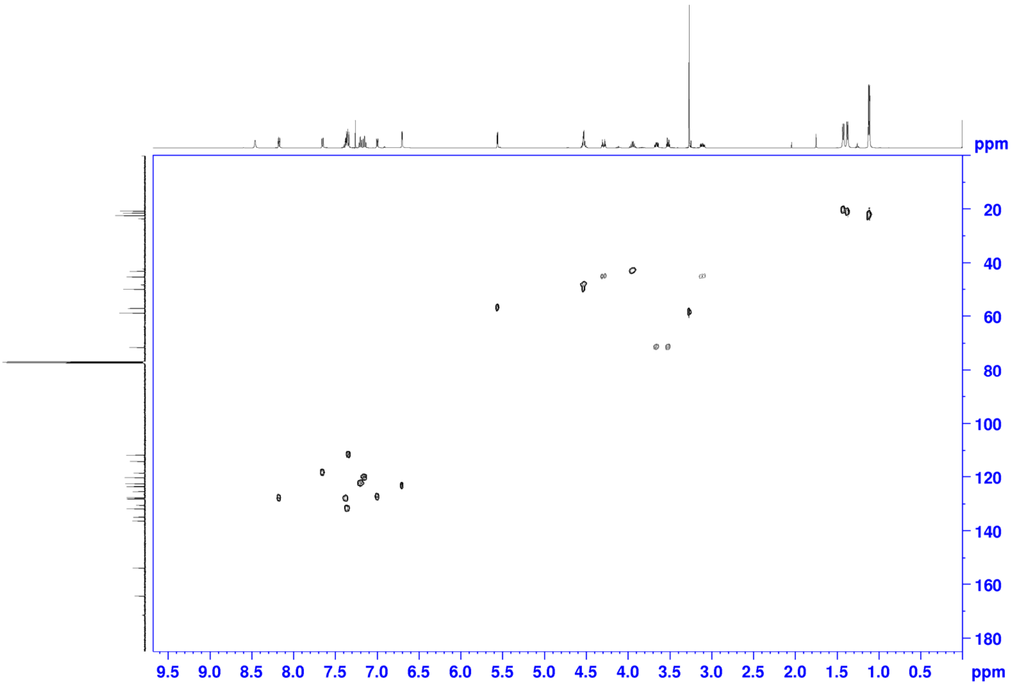

Figure S7. HSQC NMR ($CDCl_3$) of **4a** at 298 K.

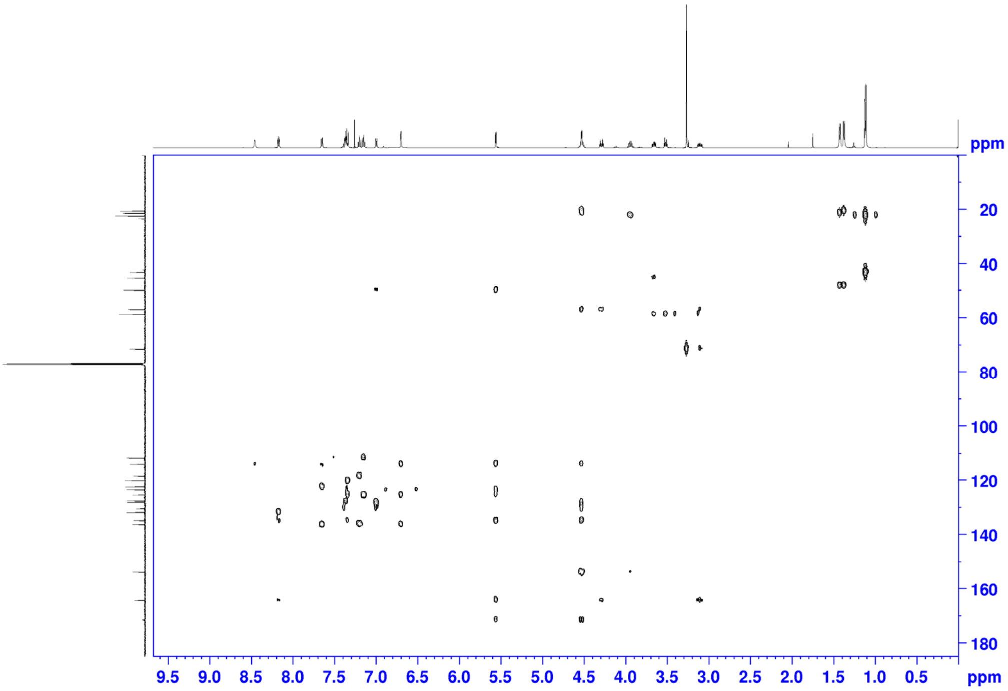

Figure S8. HMBC NMR ($CDCl_3$) of **4a** at 298 K.

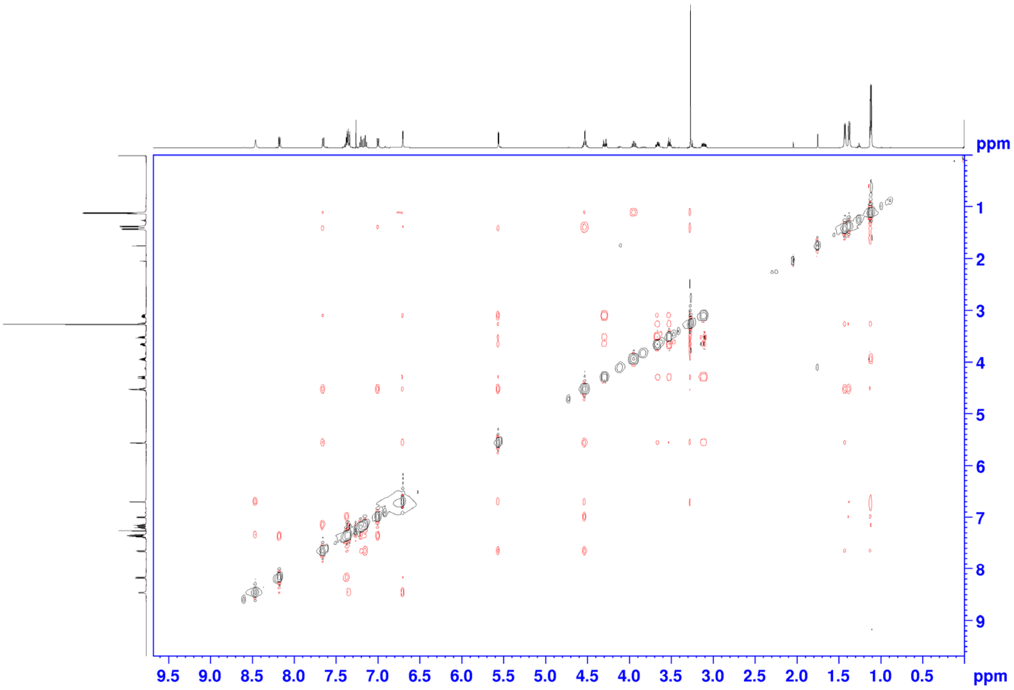

Figure S9. NOESY NMR ($CDCl_3$) of **4a** at 298 K.

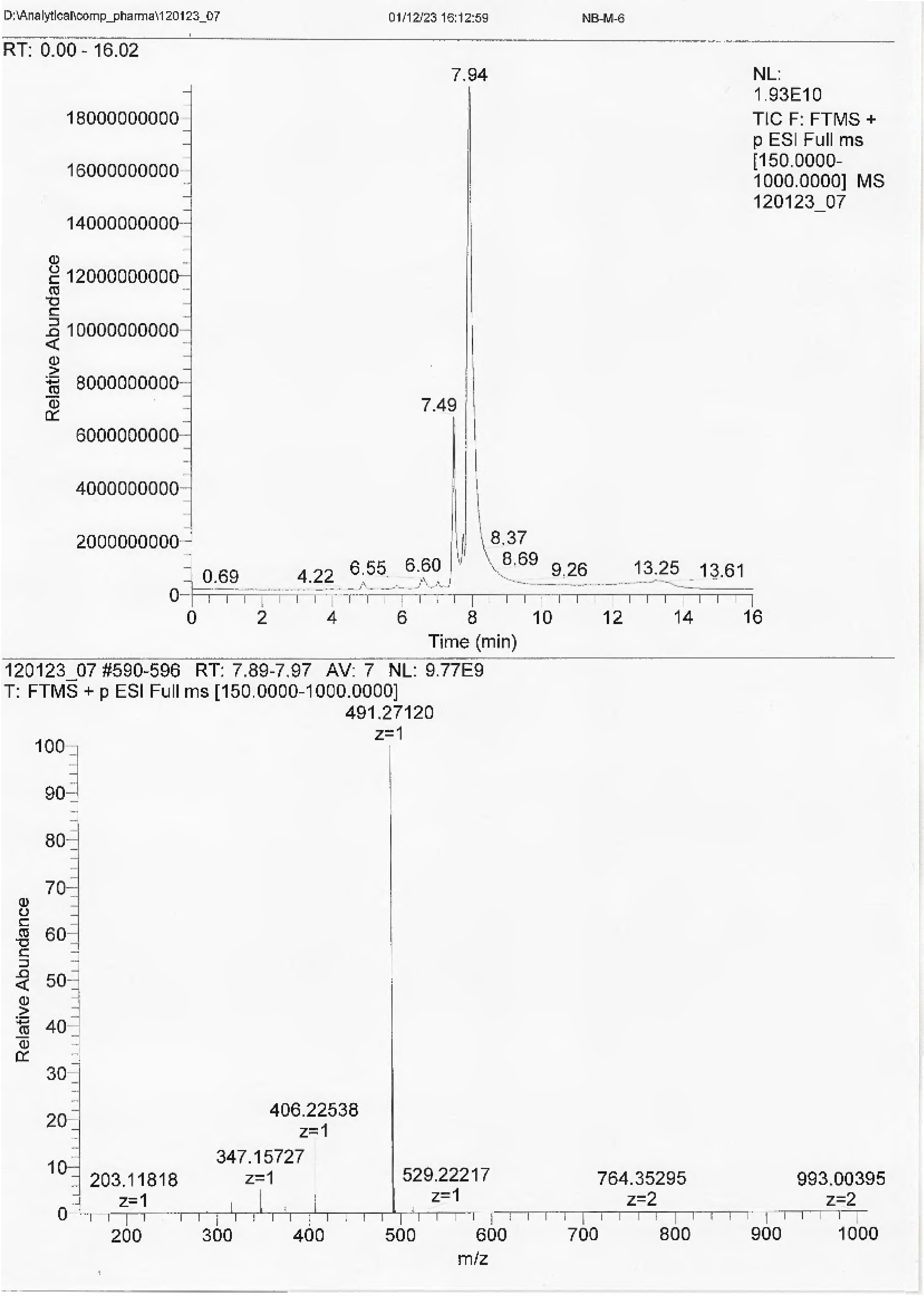

Figure S10. HRMS spectrum of **4a.**

Spectra of *Rel*-(3R,4R)-*N*-isopropyl-*N*-(isopropylcarbamoyl)-2-(2-methoxyethyl)-1-oxo-3-(1-(piperidin-1-ylmethyl)-1*H*-indol-3-yl)-1,2,3,4-tetrahydroisoquinoline-4-carboxamide (**4b**)

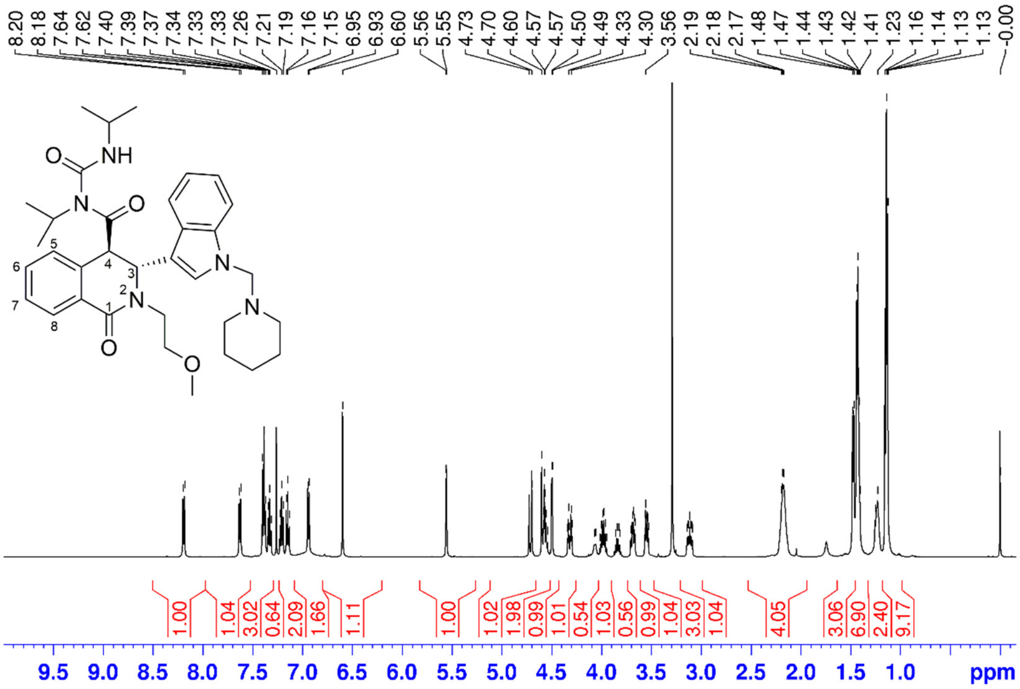

Figure S11. ^{1}H NMR ($CDCl_3$) of **4b** at 298 K.

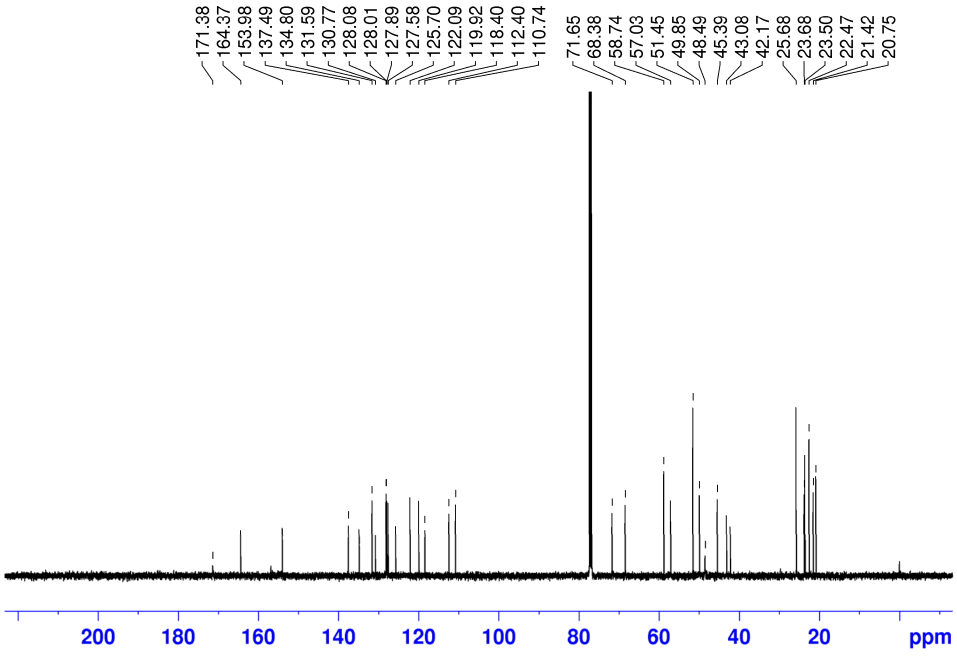

Figure S12. ^{13}C NMR ($CDCl_3$) of **4b** at 298 K.

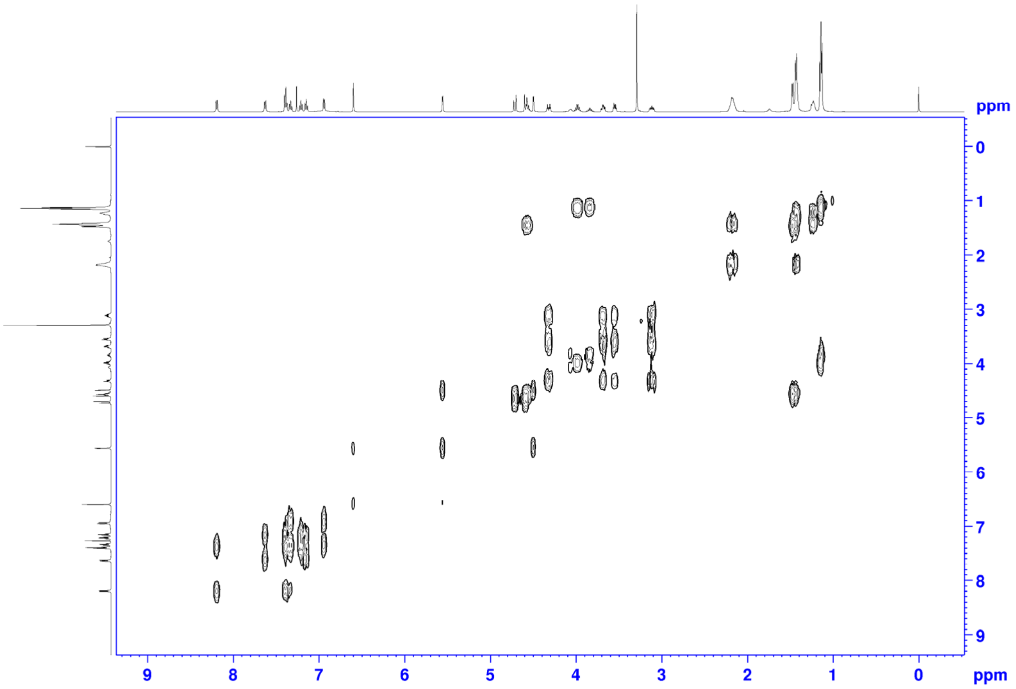

Figure S13. COSY NMR ($CDCl_3$) of **4b** at 298 K.

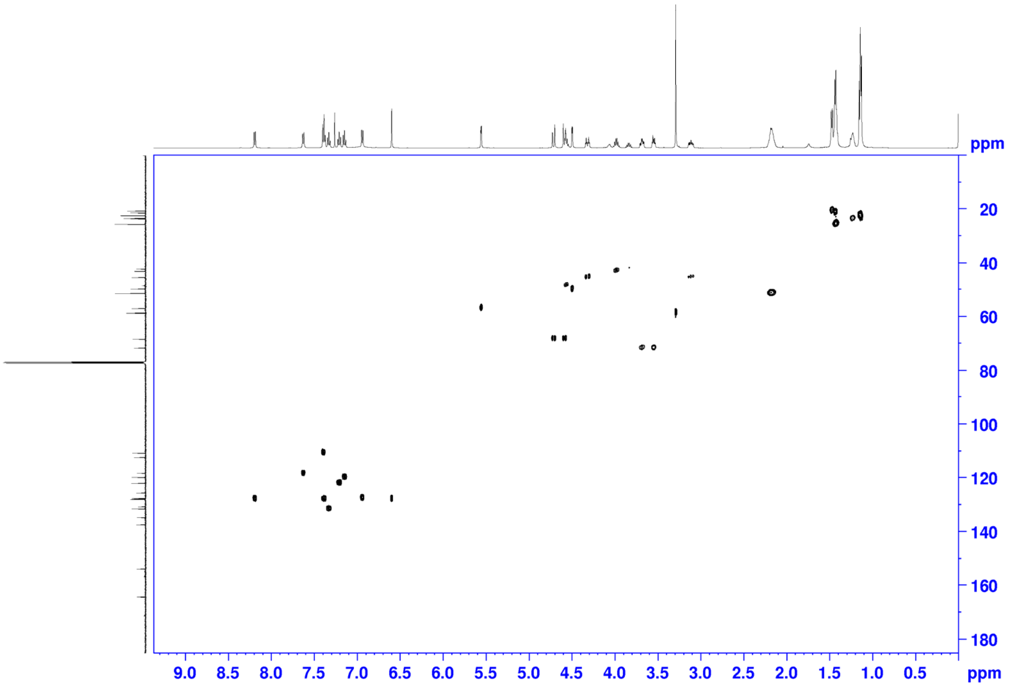

Figure S14. HSQC NMR ($CDCl_3$) of **4b** at 298 K.

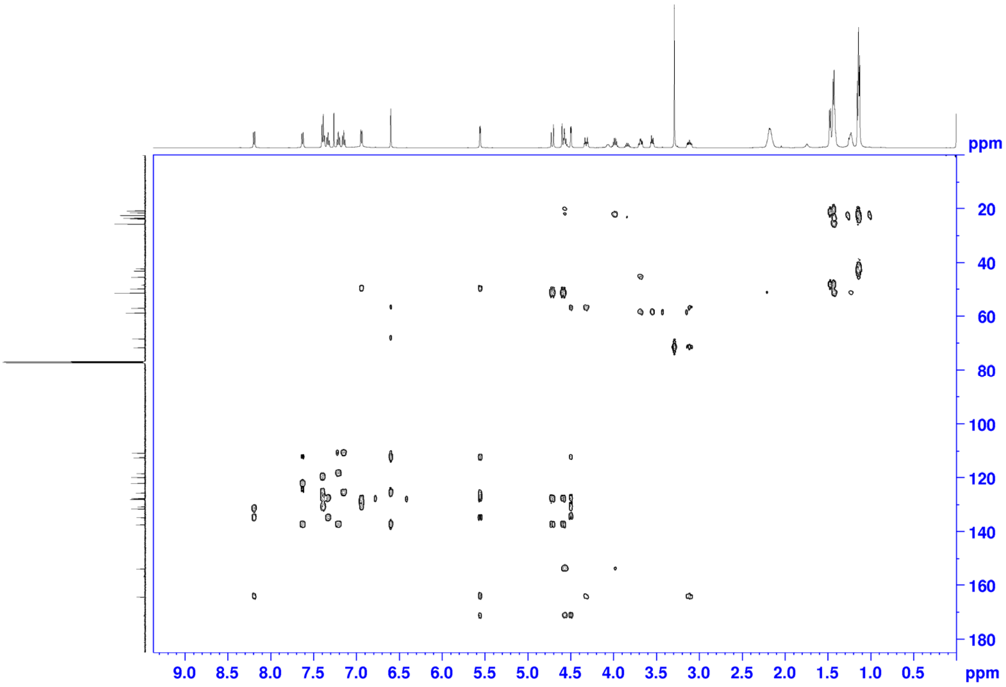

Figure S15. HMBC NMR ($CDCl_3$) of **4b** at 298 K.

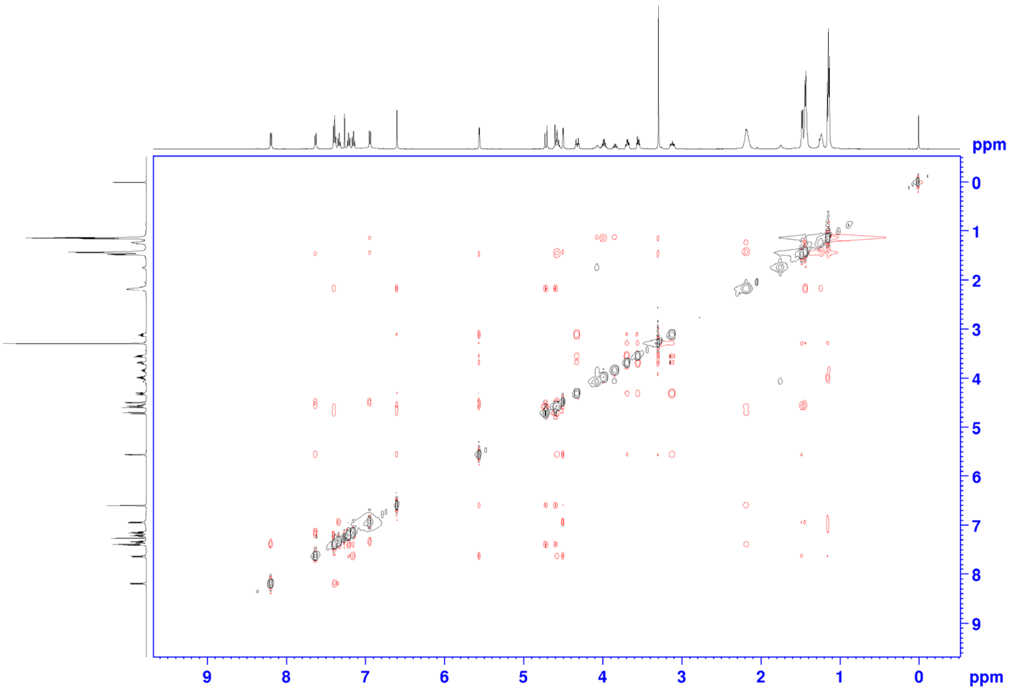

Figure S16. NOESY NMR ($CDCl_3$) of **4b** at 298 K.

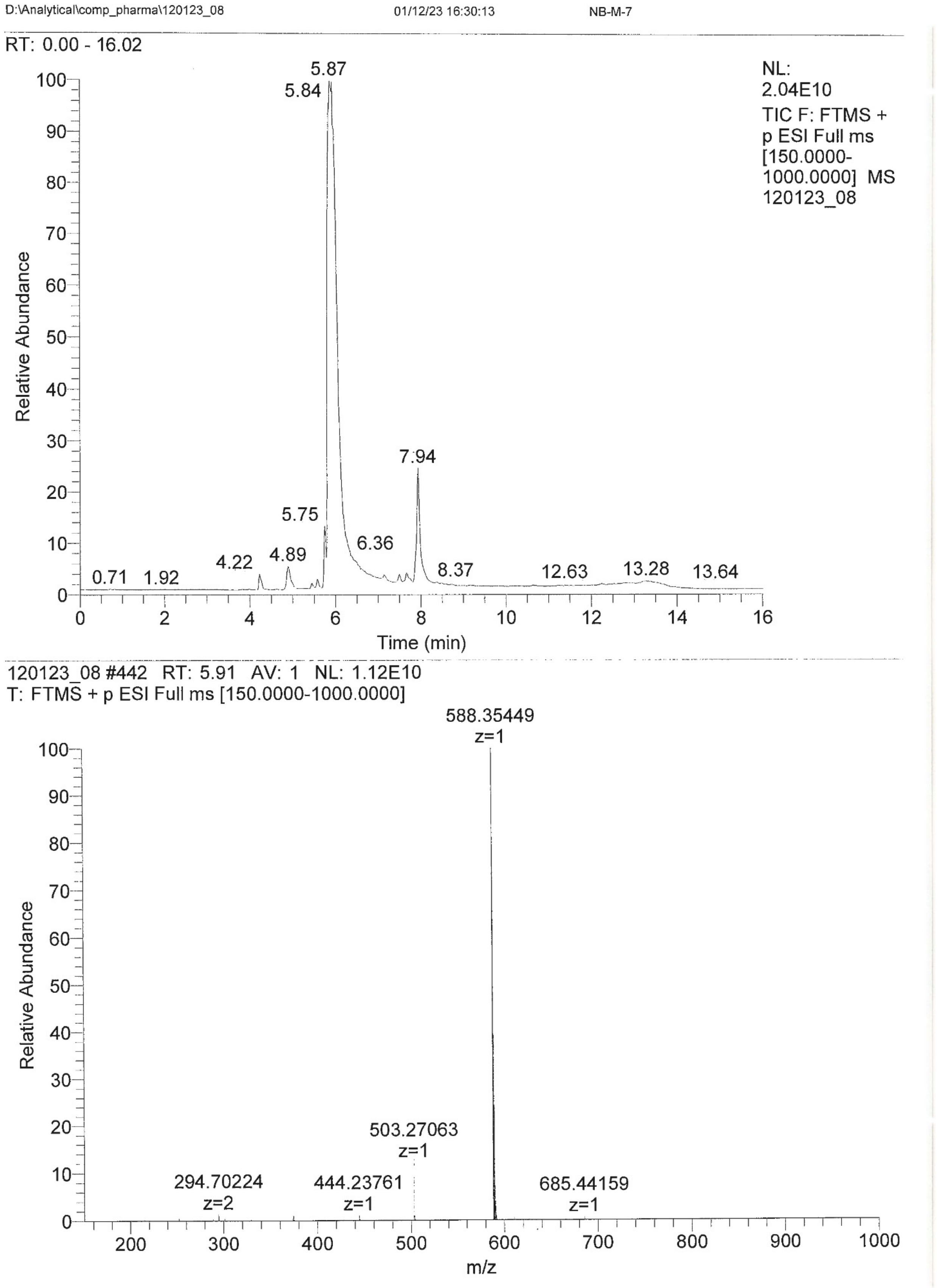

Figure S17. HRMS spectrum of **4b**.

Spectra of *Rel*-(3R,4R)-3-(1*H*-indol-3-yl)-2-(2-methoxyethyl)-4-(morpholine-4-carbonyl)-3,4-dihydroisoquinolin-1(2*H*)-one (**4c**)

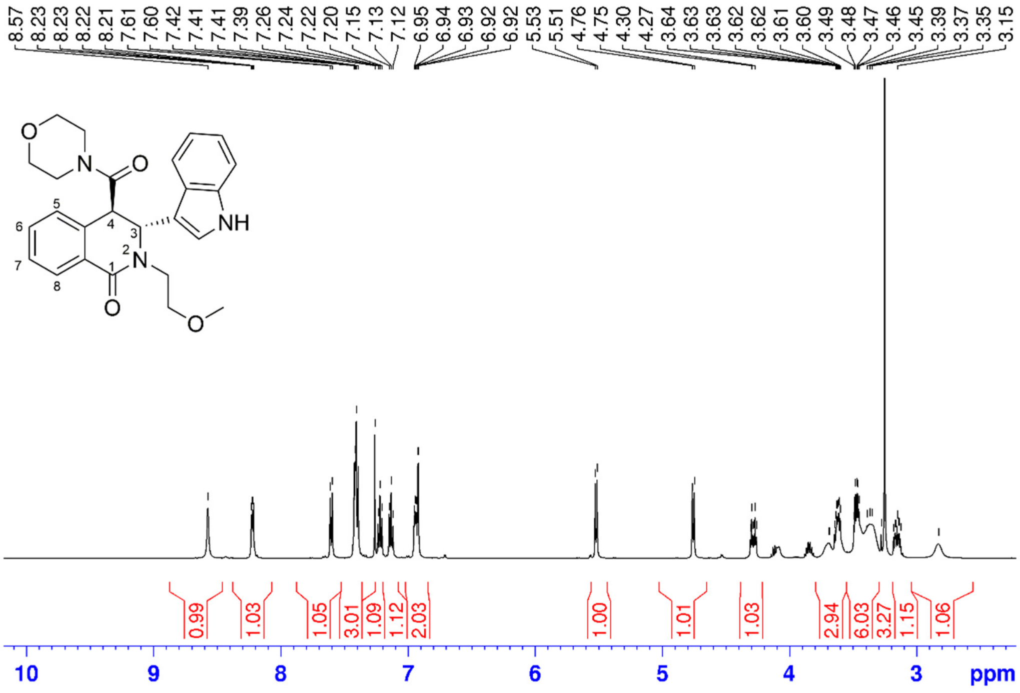

Figure S18. ^{1}H NMR ($CDCl_3$) of **4c** at 298 K.

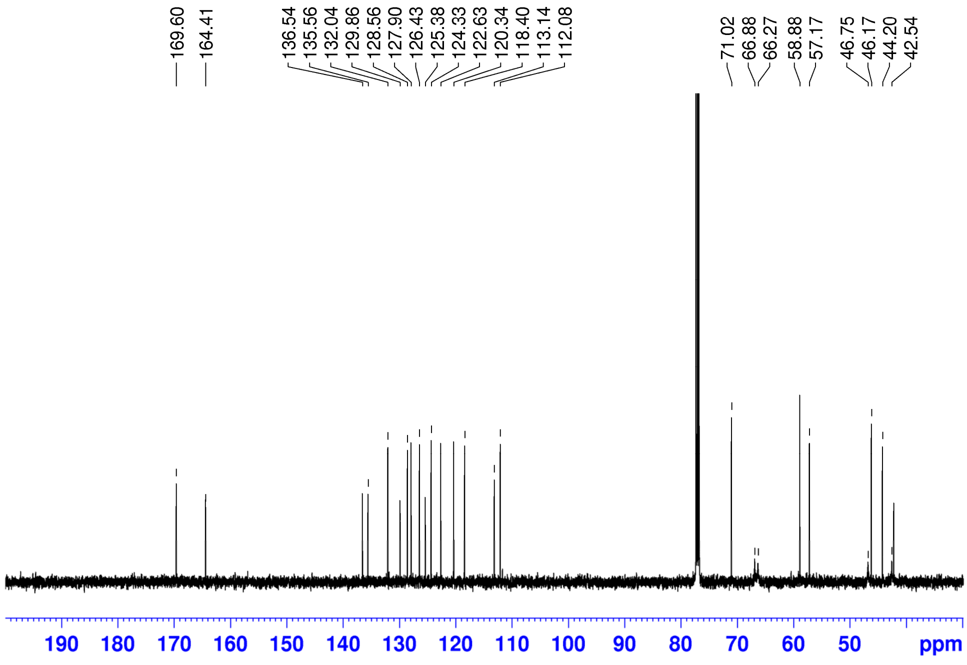

Figure S19. ^{13}C NMR NMR ($CDCl_3$) of **4c** at 298 K.

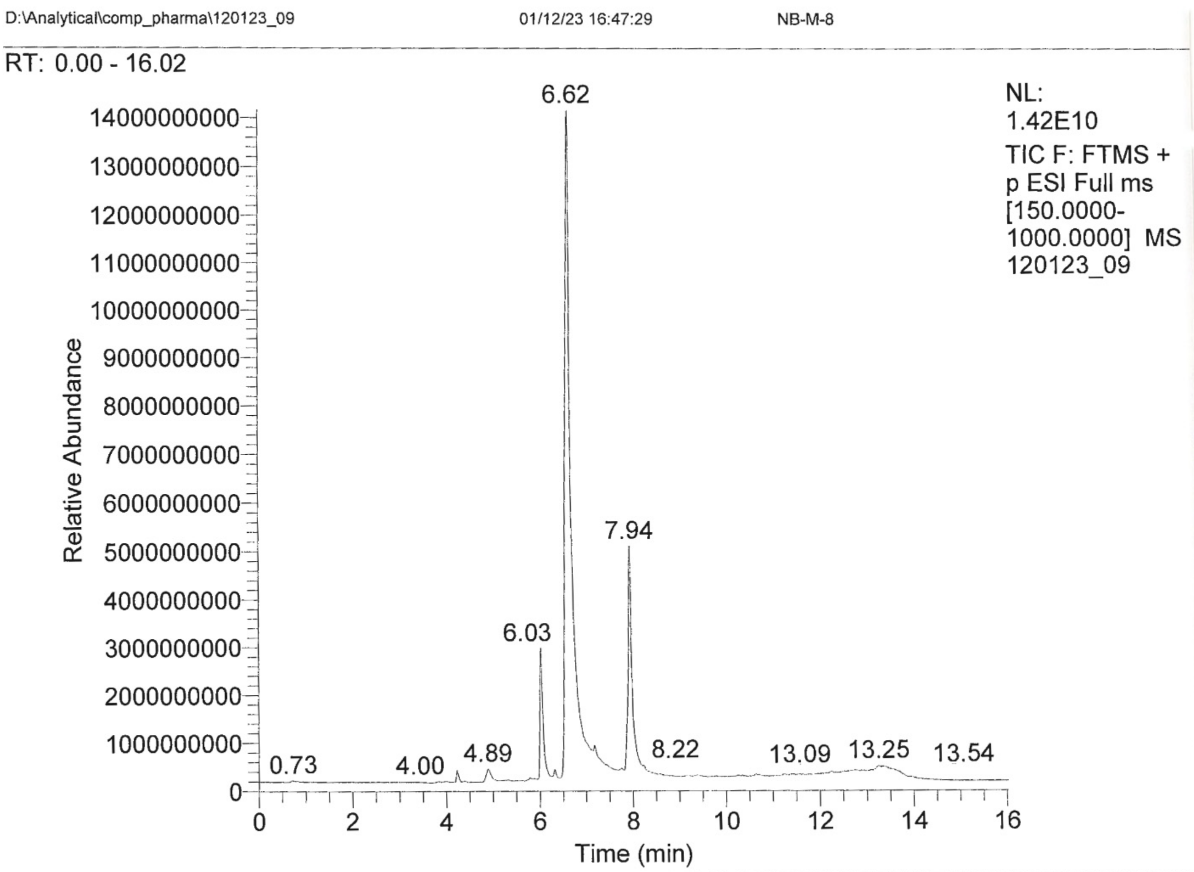

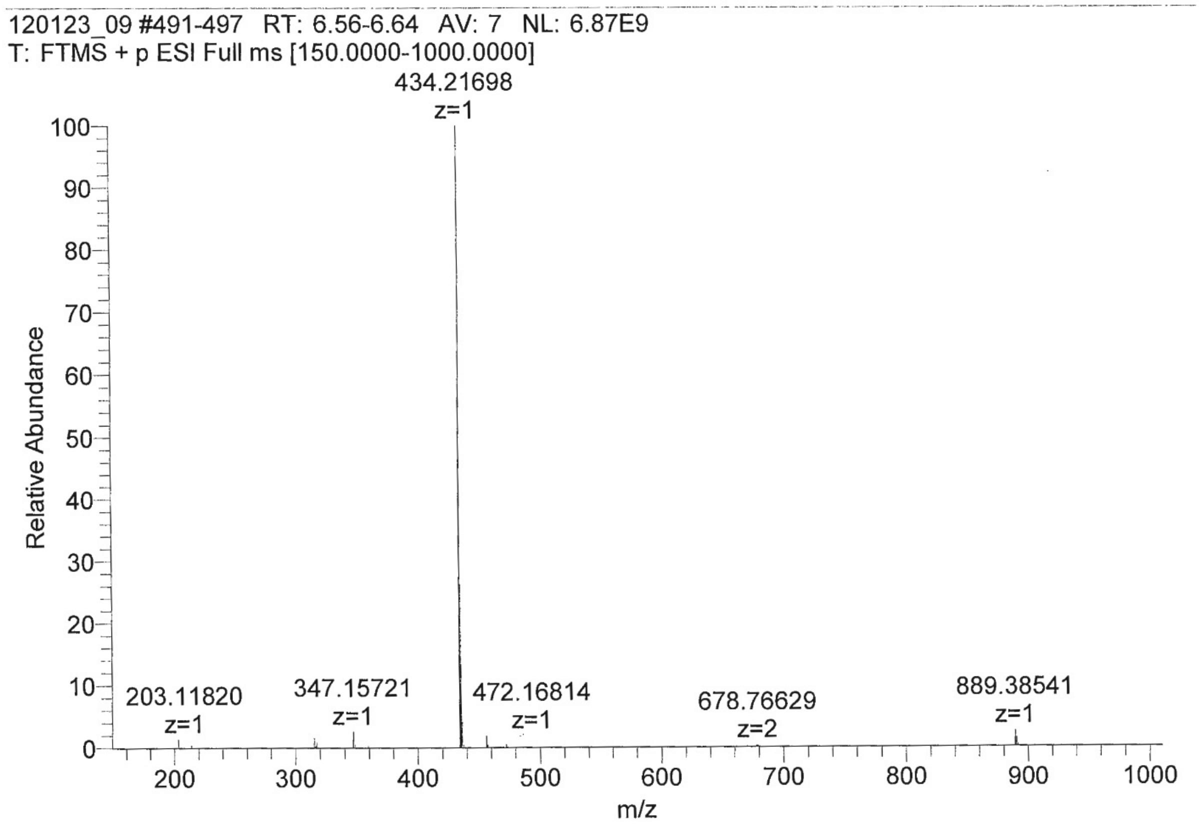

Figure S20. HRMS spectrum of **4c**.

Spectra of *Rel*-(3R,4R)-4-(1*H*-imidazole-1-carbonyl)-3-(1*H*-indol-3-yl)-2-(2-methoxyethyl)-3,4-dihydroisoquinolin-1(2*H*)-one (**4d**)

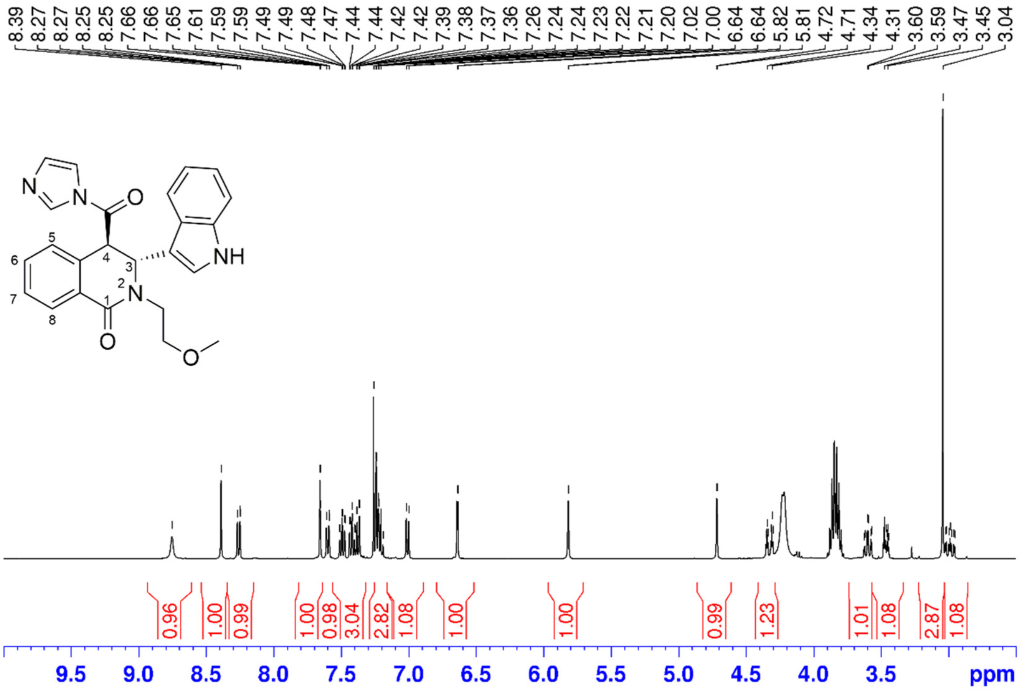

Figure S21. ^{1}H NMR (400.23 MHz, CDCl$_3$) of **4d** at 298 K.

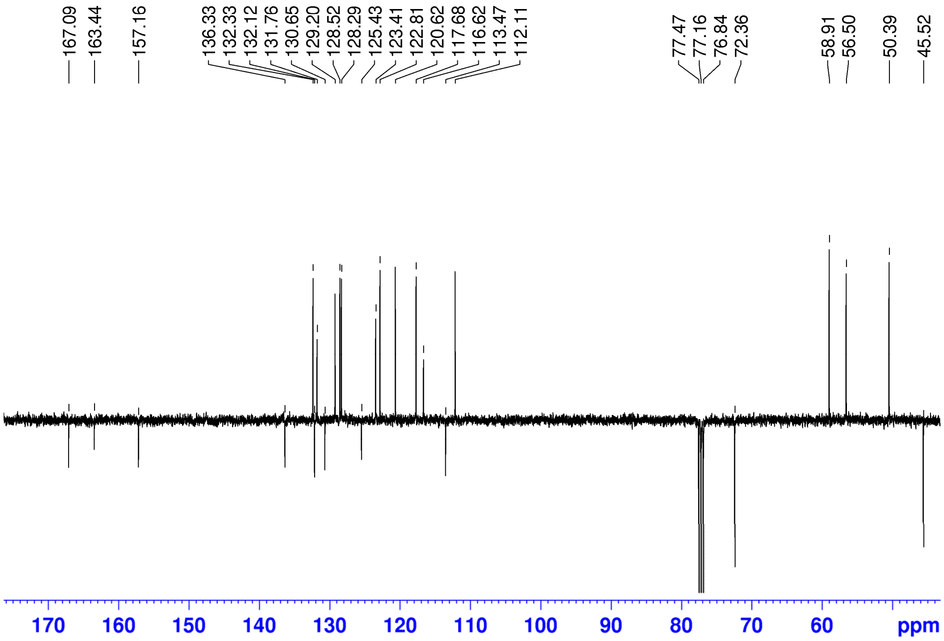

Figure S22. ^{13}C NMR using J-modulated spin-echo (100.64 MHz, CDCl$_3$) of **4d** at 298 K.

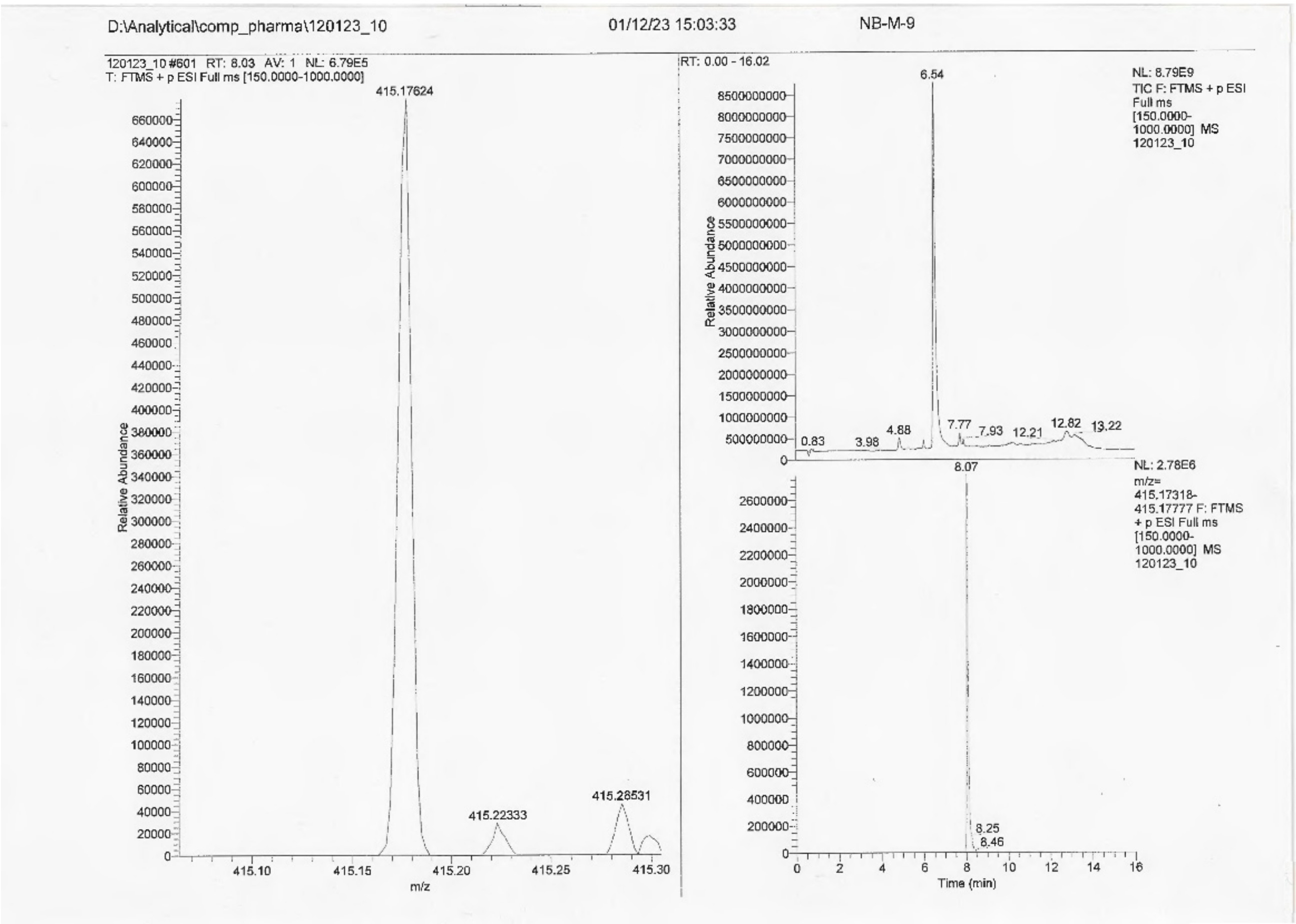

Figure S23. HRMS spectrum of **4d**.

Spectra of *Rel*-(3R,4R)-3-(1*H*-indol-3-yl)-2-(2-methoxyethyl)-4-(4-methylpiperazine-1-carbonyl)-3,4-dihydroisoquinolin-1(2*H*)-one (**4e**)

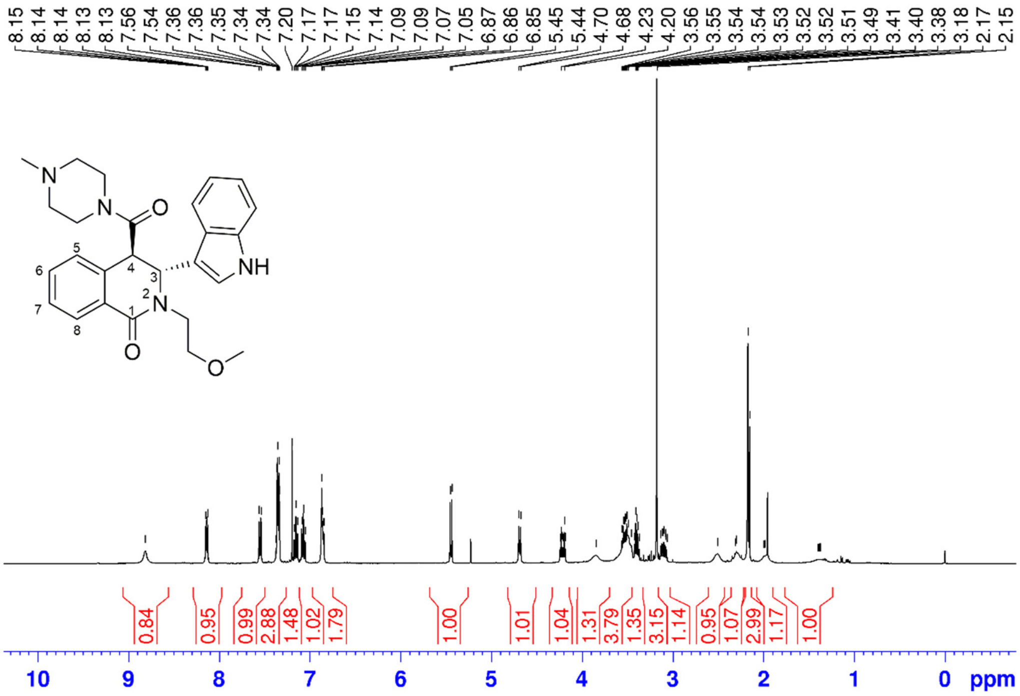

Figure S24. ^{1}H NMR (400.23 MHz, $CDCl_3$) of **4e** at 298 K.

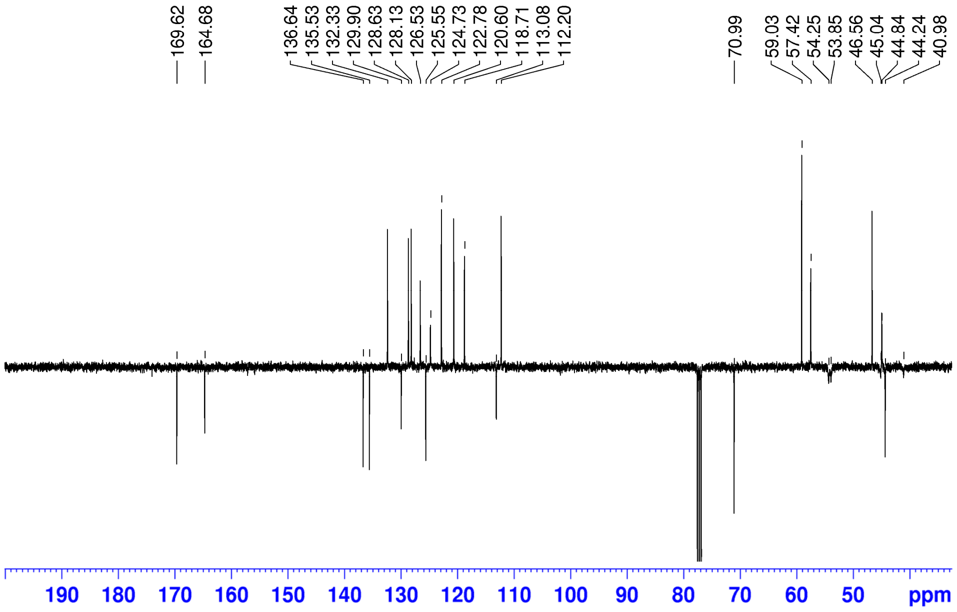

Figure S25. ^{13}C NMR using J-modulated spin-echo (100.64 MHz, $CDCl_3$) of **4e** at 298 K.

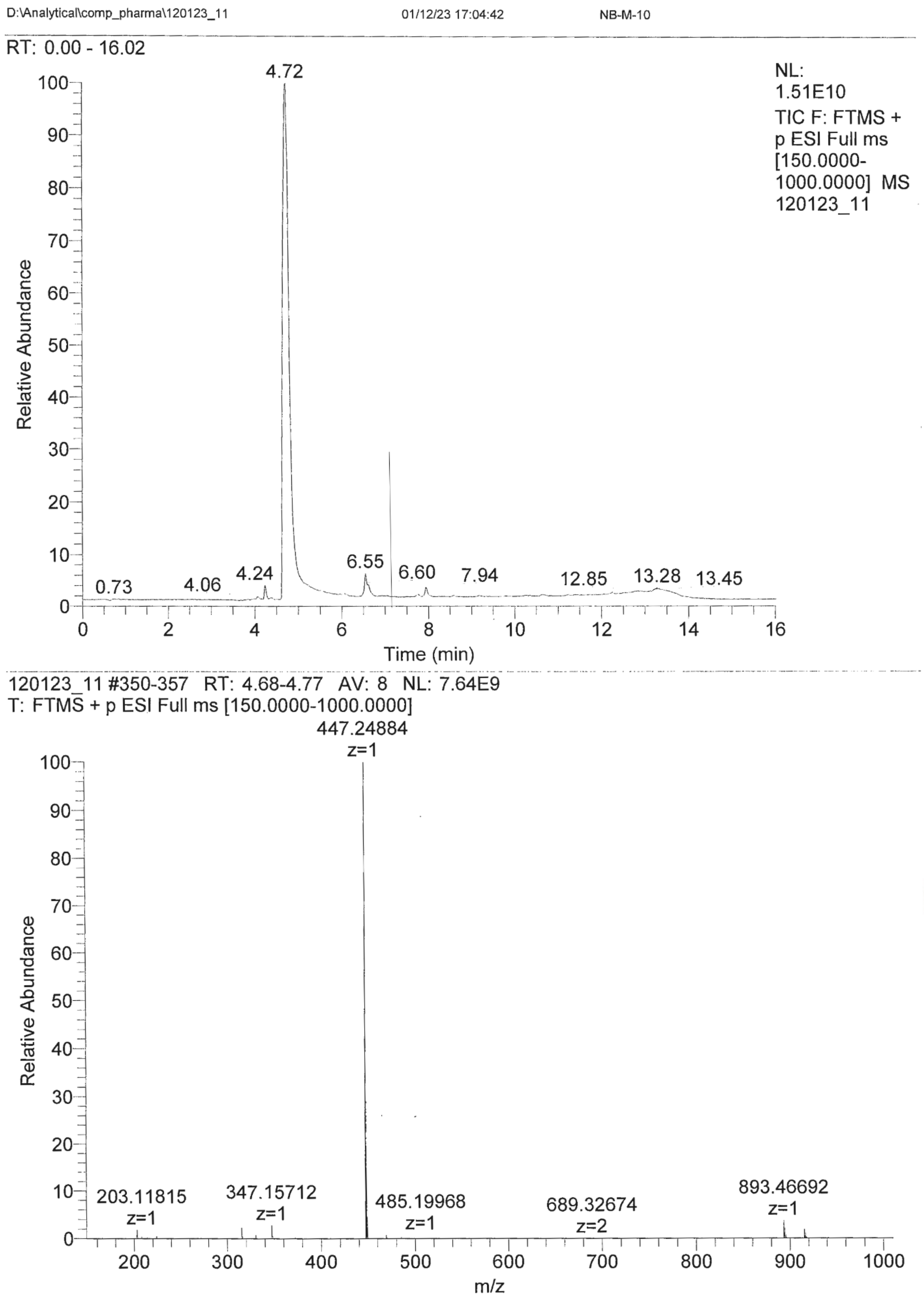

Figure S26. HRMS spectrum of **4e**.

Spectra of *Rel*-(3*R*,4*R*)- and *rel*-(3*S*,4*R*)-2-Hexyl-1-oxo-3-(pyridin-2-yl)-1,2,3,4-tetrahydroisoquinoline-4-carboxylic acids (*trans*-**6** and *cis*-**6**)

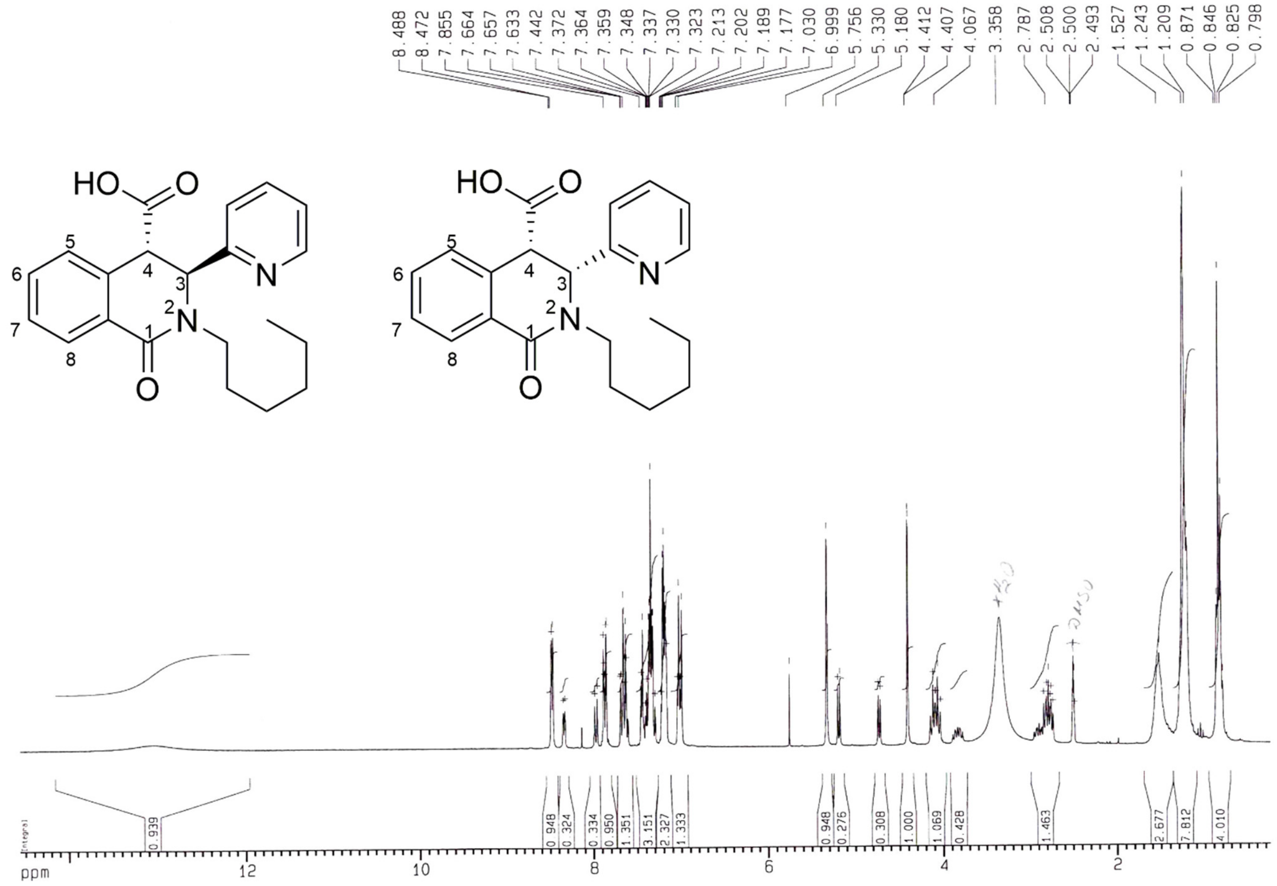

Figure S27. ^{1}H NMR (DMSO-d_6) of *trans*-**6** and *cis*-**6** at 298 K.

Spectra of *Rel*-(3*R*,4*R*)-2-Hexyl-4-(hydroxymethyl)-3-(pyridin-2-yl)-3,4-dihydroisoquinolin-1(2*H*)-one (*trans*-**8**)

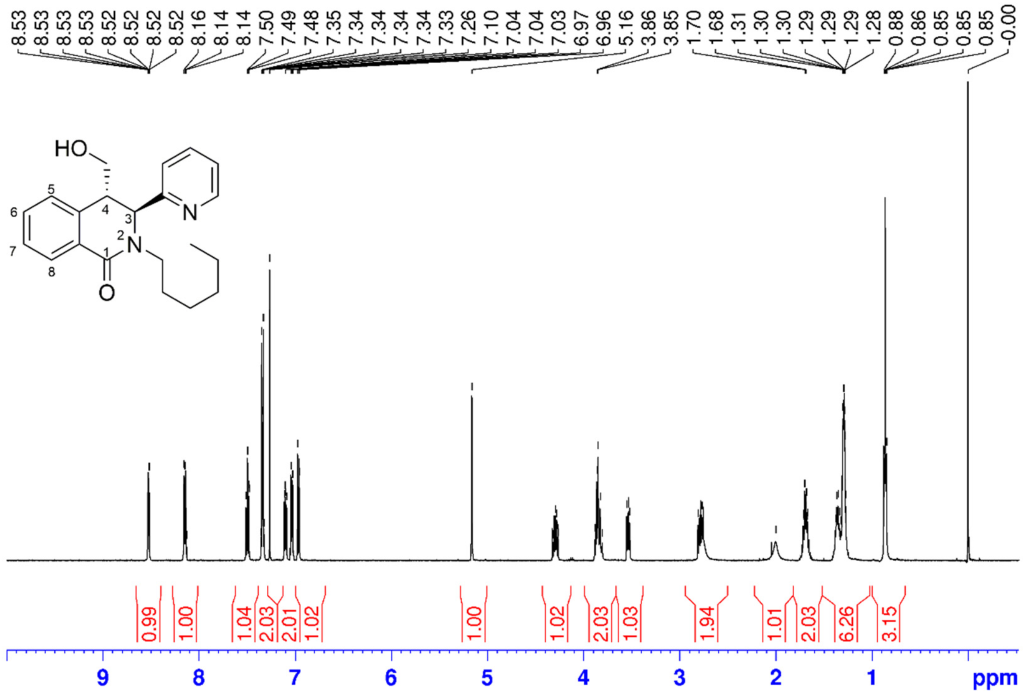

Figure S28. ^{1}H NMR (500.13 MHz, $CDCl_3$) of *trans*-**8** at 298 K.

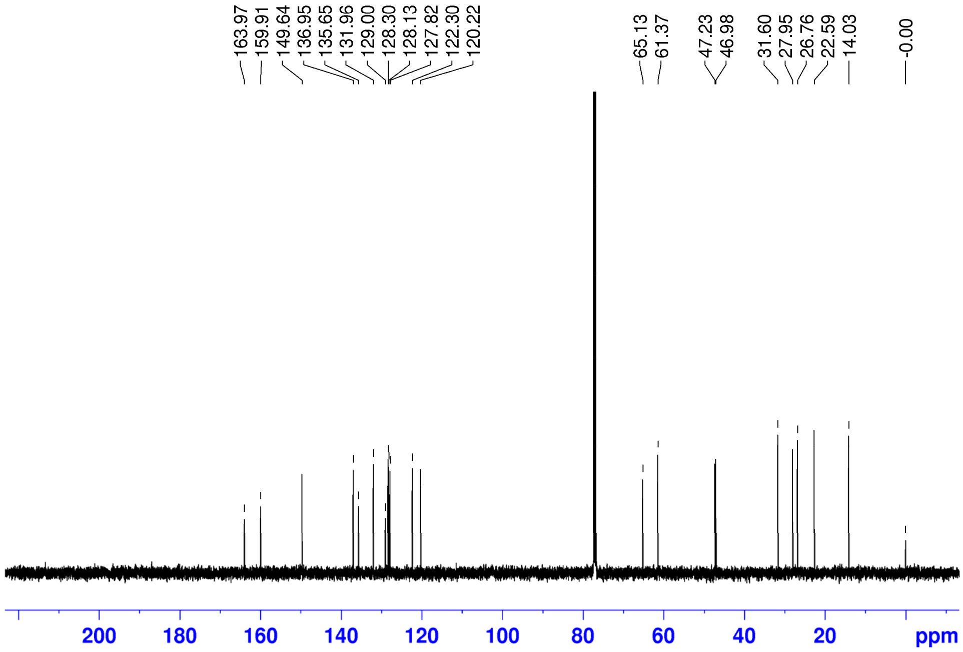

Figure S29. ^{13}C NMR ($CDCl_3$) of *trans*-**8** at 298 K.

Spectra of *Rel*-2-(((3*S*,4*R*)-2-hexyl-1-oxo-3-(pyridin-2-yl)-1,2,3,4-tetrahydroisoquinolin-4-yl)methyl)isoindoline-1,3-dione (*trans*-**9**)

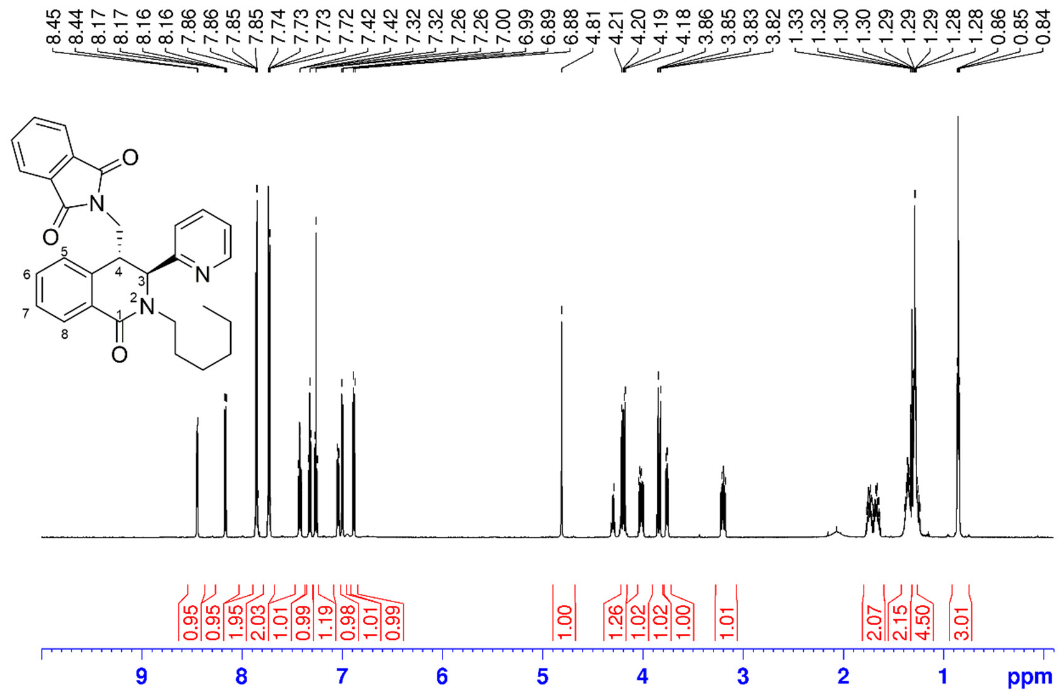

Figure S30. ^{1}H NMR (600.18 MHz, $CDCl_3$) of *trans*-**9** at 298 K.

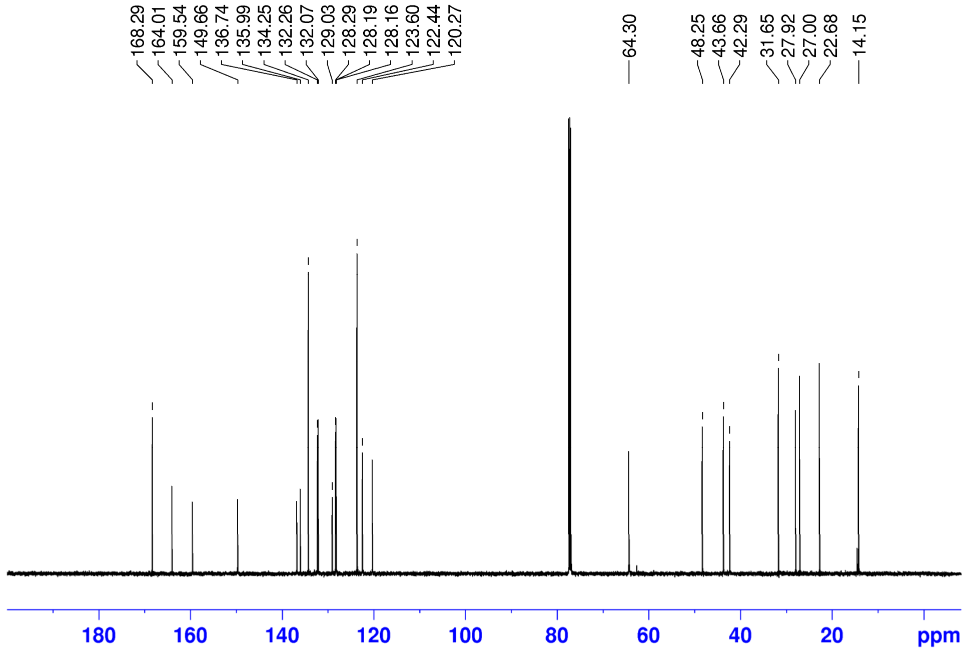

Figure S31. ^{13}C NMR ($CDCl_3$) of *trans*-**9** at 298 K.

Spectra of *Rel*-(3*R*,4*S*)-4-(Aminomethyl)-2-hexyl-3-(pyridin-2-yl)-3,4-dihydroisoquinolin-1(2*H*)-one (*trans*-**10**)

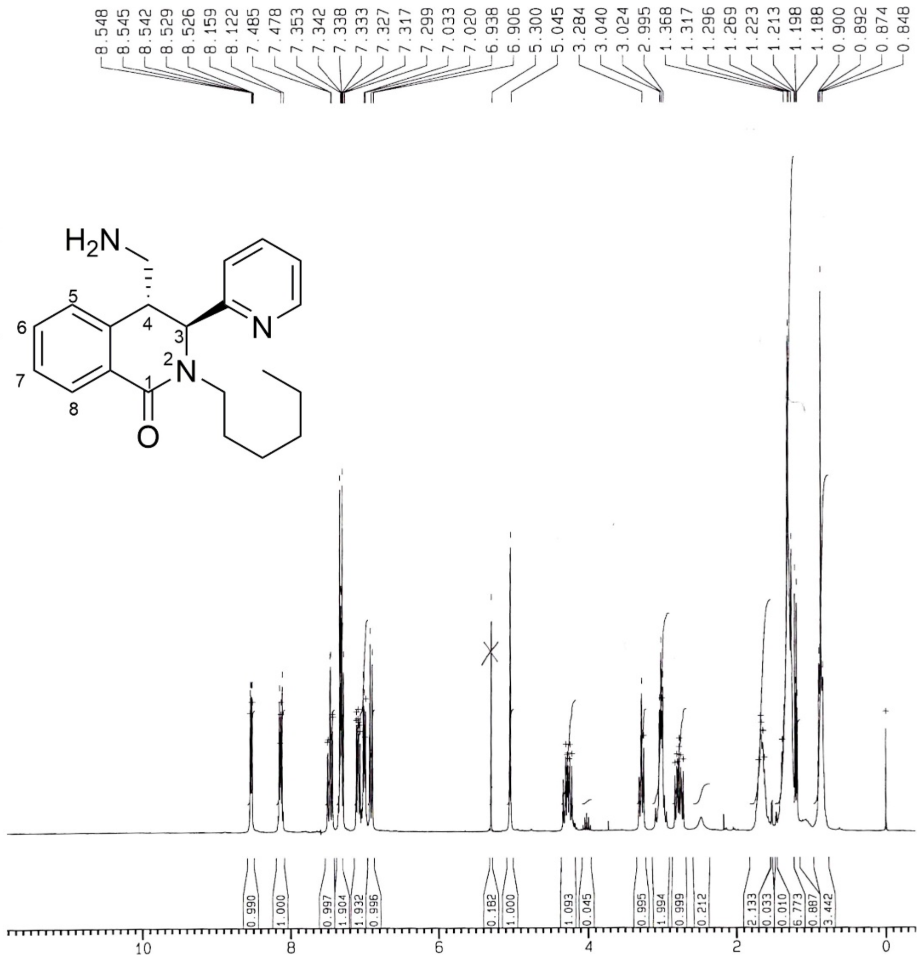

Figure S32. ^{1}H NMR (250.13 MHz, CDCl3) of *trans*-**10** at 298 K.

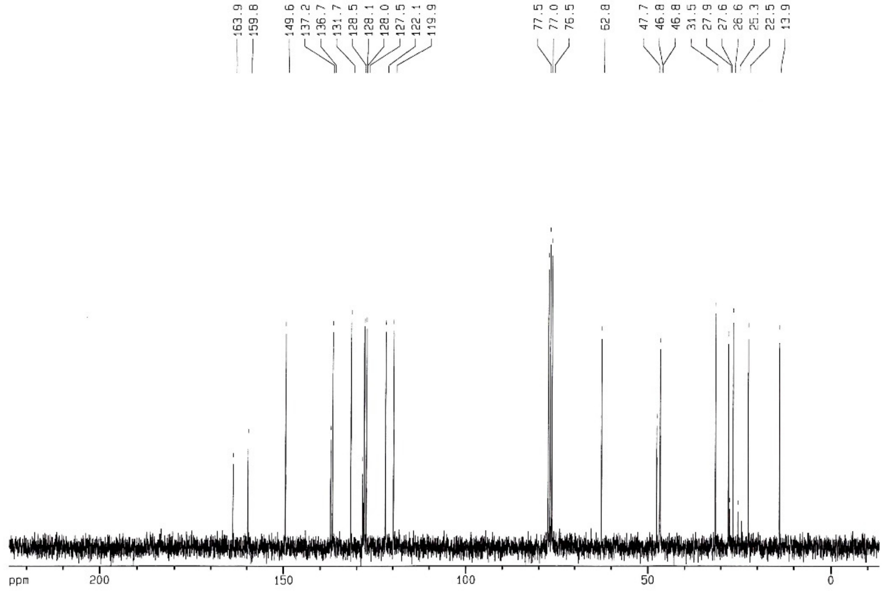

Figure S33. ^{13}C NMR ($CDCl_3$) of *trans*-**10** at 298 K.

Spectra of (*S*)-*N*-((3*R*,4*S*)-2-hexyl-1-oxo-3-(pyridin-2-yl)-1,2,3,4-tetrahydroisoquinolin-4-yl)methyl)-3-phenyl-2-(2,2,2-trifluoroacetamido)propanamide and (*S*)-*N*-((3*S*,4*R*)-2-hexyl-1-oxo-3-(pyridin-2-yl)-1,2,3,4-tetrahydroisoquinolin-4-yl)methyl)-3-phenyl-2-(2,2,2-trifluoroacetamido)propanamide (*trans*-**11a**+*trans*-**11b**)

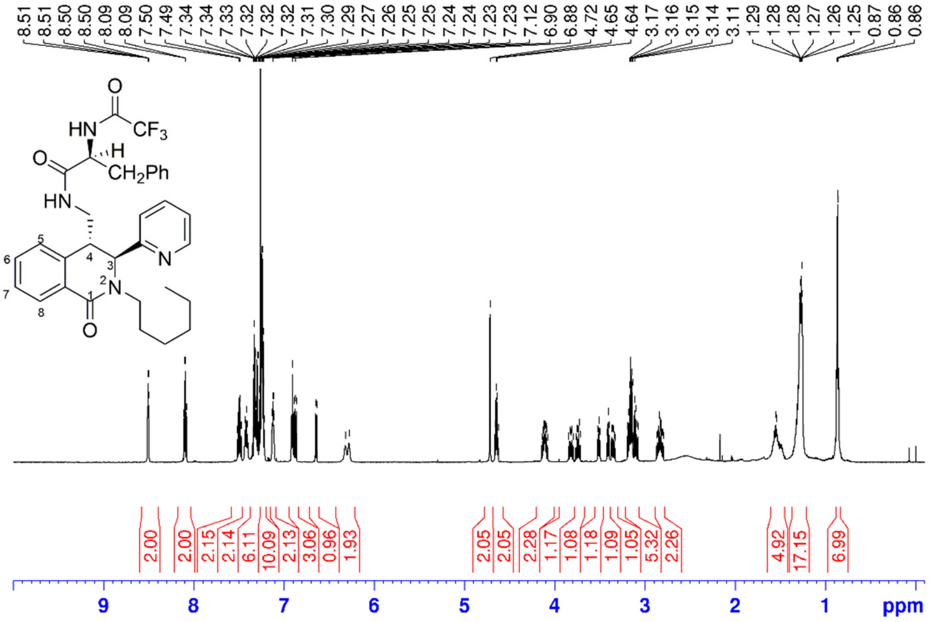

Figure S34. ¹H NMR (600.18 MHz, CDCl3) of *trans*-**11a**+*trans*-**11b** at 298 K.

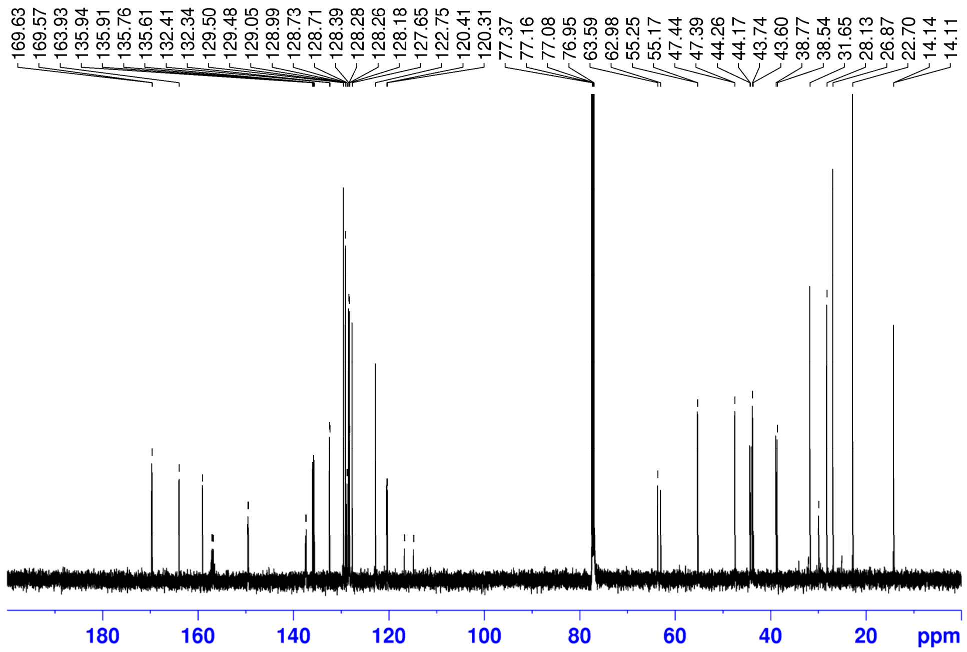

Figure S35. ^{13}C NMR ($CDCl_3$) of *trans*-**11a**+*trans*-**11b** at 298 K.

Spectra of *Tert*-Butyl (*S*)-2-(((3*R*,4*S*)-2-hexyl-1-oxo-3-(pyridin-2-yl)-1,2,3,4-tetrahydroisoquinolin-4-yl)methyl)carbamoyl)pyrrolidine-1-carboxylate and *tert*-butyl (*S*)-2-(((3*S*,4*R*)-2-hexyl-1-oxo-3-(pyridin-2-yl)-1,2,3,4-tetrahydroisoquinolin-4-yl)methyl)carbamoyl)pyrrolidine-1-carboxylate (*trans*-**12a**+trans-**12b**)

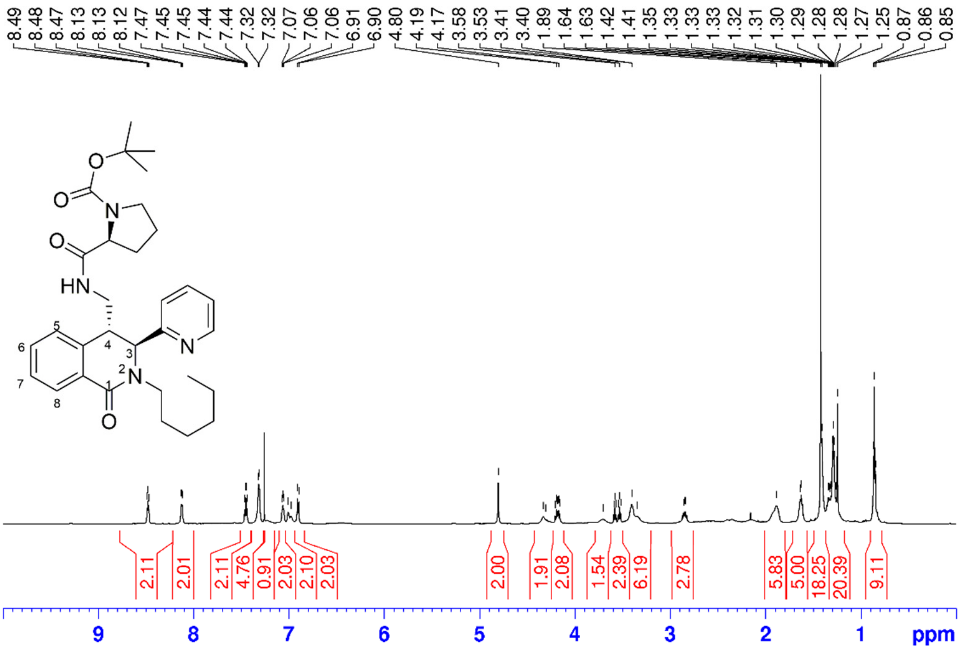

Figure S36. ^{1}H NMR (250.13 MHz, CDCl3) of *trans*-**12a**+*trans*-**12b** at 298 K.

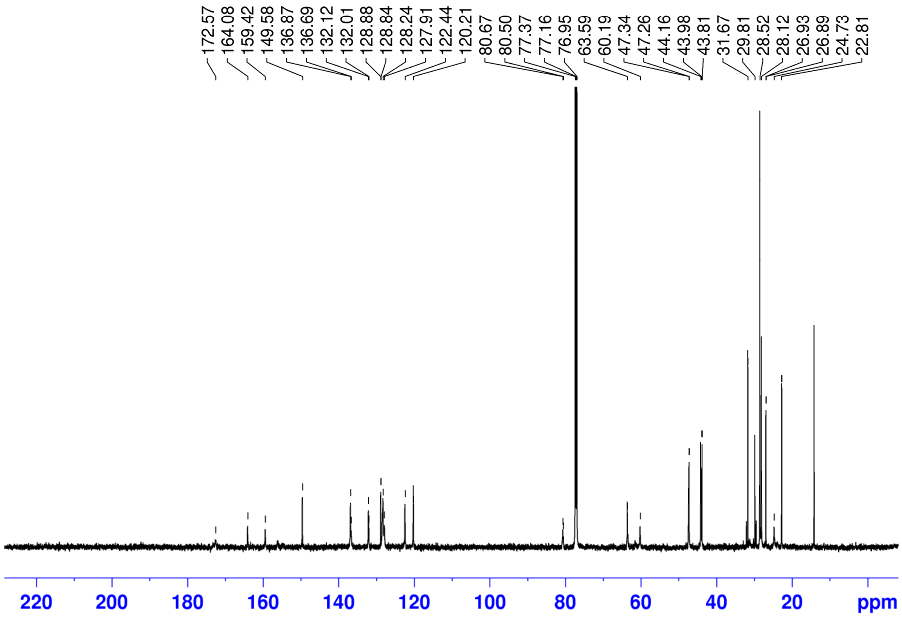

Figure S37. ^{13}C NMR ($CDCl_3$) of *trans*-**12a**+*trans*-**12b** at 298 K.

(*S*)-*N*-(((3*R*,4*S*)-2-hexyl-1-oxo-3-(pyridin-2-yl)-1,2,3,4-tetrahydroisoquinolin-4-yl)methyl)-4-(methylthio)-2-(2,2,2-trifluoroacetamido)butanamide and (*S*)-*N*-(((3*S*,4*R*)-2-hexyl-1-oxo-3-(pyridin-2-yl)-1,2,3,4-tetrahydroisoquinolin-4-yl)methyl)-4-(methylthio)-2-(2,2,2-trifluoro-acetamido)butanamide (*trans*-**13a**+*trans*-**13b**)

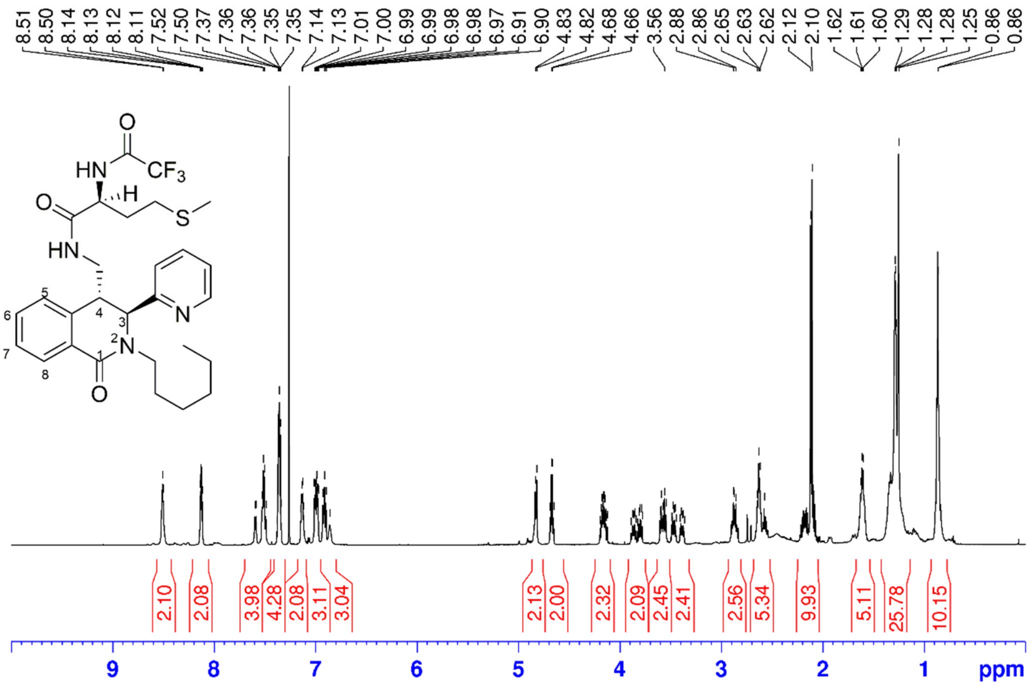

Figure S38. ¹H NMR (250.13 MHz, CDCl3) of *trans*-**13a**+*trans*-**13b** at 298 K.

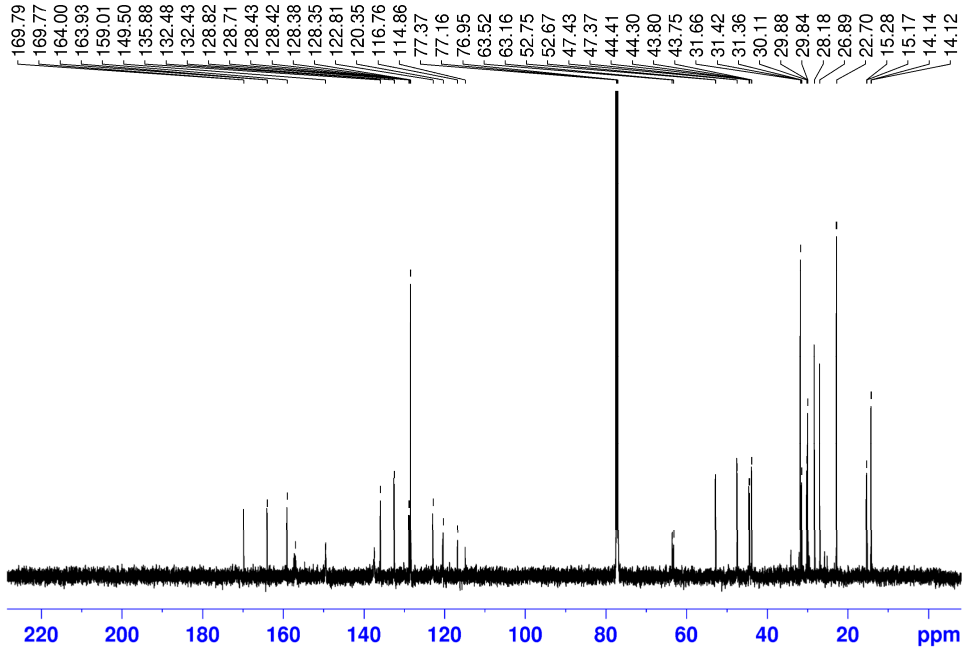

Figure S39. ^{13}C NMR ($CDCl_3$) of *trans*-**13a**+*trans*-**13b** at 298 K.

Optimized geometry at PCM/M06-2X/6-31+G(d,p) level of theory (solvent dichloroethane) of trans-6a′

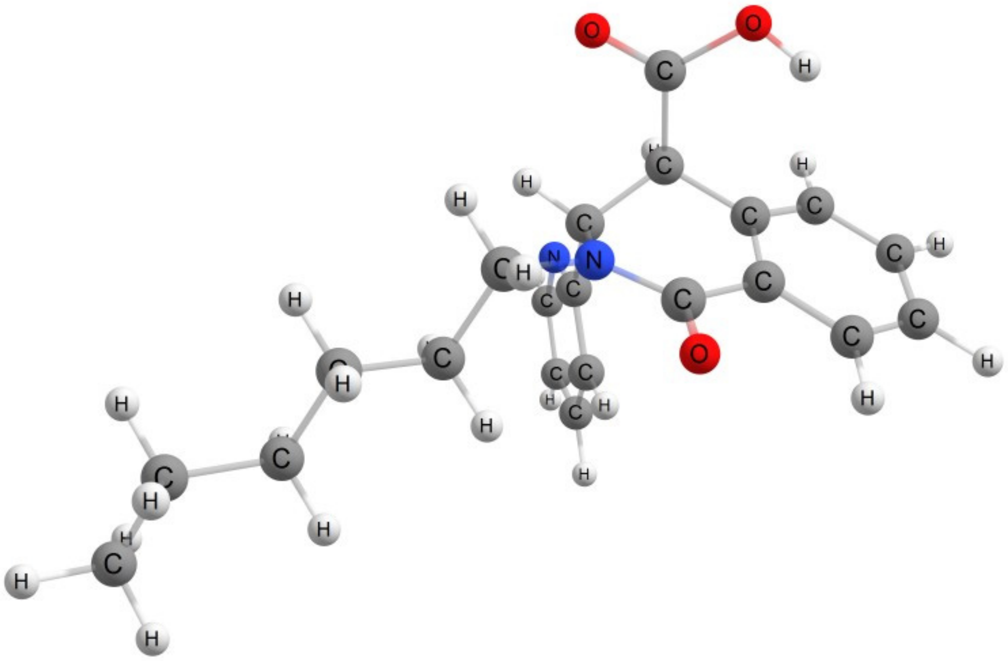

Figure S40. Optimized geometry at PCM/M06-2X/6-31+G(d,p) level of theory (solvent dichloroethane) of trans-6a′
